# 3D Microfluidic model for evaluating immunotherapy efficacy by tracking dendritic cell behaviour toward tumor cells

**DOI:** 10.1038/s41598-017-01013-x

**Published:** 2017-04-24

**Authors:** Stefania Parlato, Adele De Ninno, Rosa Molfetta, Elena Toschi, Debora Salerno, Arianna Mencattini, Giulia Romagnoli, Alessandra Fragale, Lorenzo Roccazzello, Maria Buoncervello, Irene Canini, Enrico Bentivegna, Mario Falchi, Francesca Romana Bertani, Annamaria Gerardino, Eugenio Martinelli, Corrado Natale, Rossella Paolini, Luca Businaro, Lucia Gabriele

**Affiliations:** 1 Department of Oncology and Molecular Medicine, Istituto Superiore di Sanità, 00161 Rome, Italy; 2 Department of Civil Engineering and Informatic Science, University of Rome Tor Vergata, 00133 Rome, Italy; 3 Department of Molecular Medicine, Istituto Pasteur-Fondazione Cenci Bolognetti, “Sapienza” University of Rome, 00161 Rome, Italy; 4 Institute for Photonics and Nanotechnology, Italian National Research Council, 00156 Rome, Italy; 5 Center for Life Nano Science@Sapienza, Istituto Italiano di Tecnologia, 00161 Rome, Italy; 6 Department of Electronic Engineering, University of Rome Tor Vergata, 00133 Rome, Italy; 7 National AIDS Center, Istituto Superiore di Sanità, 00161 Rome, Italy

## Abstract

Immunotherapy efficacy relies on the crosstalk within the tumor microenvironment between cancer and dendritic cells (DCs) resulting in the induction of a potent and effective antitumor response. DCs have the specific role of recognizing cancer cells, taking up tumor antigens (Ags) and then migrating to lymph nodes for Ag (cross)-presentation to naïve T cells. Interferon-α-conditioned DCs (IFN-DCs) exhibit marked phagocytic activity and the special ability of inducing Ag-specific T-cell response. Here, we have developed a novel microfluidic platform recreating tightly interconnected cancer and immune systems with specific 3D environmental properties, for tracking human DC behaviour toward tumor cells. By combining our microfluidic platform with advanced microscopy and a revised cell tracking analysis algorithm, it was possible to evaluate the guided efficient motion of IFN-DCs toward drug-treated cancer cells and the succeeding phagocytosis events. Overall, this platform allowed the dissection of IFN-DC-cancer cell interactions within 3D tumor spaces, with the discovery of major underlying factors such as CXCR4 involvement and underscored its potential as an innovative tool to assess the efficacy of immunotherapeutic approaches.

## Introduction

Immunotherapy relies on the use of therapeutic agents that are able to potentiate immune effector mechanisms also inside the tumor microenvironment (TME)^1^. In this context, the adjuvant capacity of dendritic cells (DCs) is crucial in determining the success of these treatments, especially in case of poorly immunogenic tumors^2^. DCs have the capability to scan the microenvironment and to capture and present antigens (Ag) to lymphocytes to generate an antitumor immune response^3^. Since the balance between stimulatory and suppressive signals within the TME determines DC functions, the prevalence of immunosuppressive inflammation hampers the antitumor activities of these cells and the development of an efficient antitumor immunity^2^. Conversely, tumor treatment with agents that favour the release of immunogenic signals by dying cancer cells promotes the adjuvant capability of DCs to induce antitumor responses^4–6^. Recent studies have demonstrated that epigenetic therapies are able to determine tumor lysis and re-establish endogenous immune recognition thus enhancing the antitumor immune response. In addition, epigenetic drugs and immunotherapy have been proposed as a particularly promising combination to combat cancer^7^. Along this line, we have recently reported that the combination of IFN-α (I) and epigenetic drugs, such as the DNA methyltransferase inhibitor (DNMTi) 5-azacitidine and the histone deacetylase inhibitor (HDACi) romidepsin (R) represents an efficacious antitumor treatment with a high potential to induce immunogenic apoptosis of colorectal cancer (CRC) cells^8^. Upon phagocytosis of dying cancer cells, DCs fulfil their primary role by processing and presenting tumor Ags to CD4^+^ T helper cells, while some subsets of DCs possess the capability to cross-present tumor Ags to CD8^+^ T cells, and thus stimulate the effector cells of the antitumor response^9^. These peculiar DC functions evoking antitumor immunity have been exploited in several DC-based therapeutic approaches. In our laboratory, we developed IFN-α-conditioned DCs (IFN-DCs) as promising candidates for therapeutic cancer vaccines^10^. These cells possess superior properties in Ag uptake and induction of both CD4^+^ T helper lymphocytes and CD8^+^ cytotoxic T cells and resemble naturally occurring DCs^11–13^. It is important to note that the superior functional activities of IFN-DCs, as well as the rapid acquisition of their potent migratory ability, may also depend on the expression of chemokine receptors^14^. Data from a pilot clinical study indicate that in patients with advanced melanoma, intratumoral injection of IFN-DCs after dacarbazine treatment activates antitumor immunity confirming the high capability of these cells to fulfil their functions upon Ag release *in situ* ^15^.

Microfluidic models represent a new frontier for studying the complex interactions occurring within organ microenvironments in both physiologic and pathologic conditions^16, 17^. The recent advances in both cell biology and microengineering have allowed the development of the innovative organ-on-chip approach, which is able to recapitulate *in vivo* biological microenvironments suitable for studying complex functions, such as cell-cell interactions and dynamic drug stimuli^18, 19^. This enormous potential relies first on the recreation of complex 3D spaces characterized by both physical and biochemical cues closely mimicking the *in vivo* microenvironments^20^. Importantly, microfluidic platforms are able to reproduce cell confinement, a parameter imposed on cell movement in the interstitial space of tissues, which is totally absent in 2D assays. This confinement is essential for studying the behaviour of motile cells such as immune and cancer cells^21^. The coordinated integration of a microfluidic assay, advanced microscopy and computational modelling enables the observation of single events as part of the complex biological processes ultimately leading to define the physiopathological responses^22, 23^. These breakthrough innovations have allowed the study of cancer-immune interactions as well as immunotherapeutic treatments using microfluidic platforms^24^. In oncology, microfluidic models have been widely used to study the metastatic potential of cancer cells^25, 26^. In the past few years, our group exploited the microfluidic approach to investigate in real time the interactions between immune and cancer cells occurring during an IRF-8-deficient antitumor immune response^27, 28^. This approach provides a new method to investigate these events also under therapeutic treatments^29^. However, one major challenge is the proper reconstruction of tumor and immune systems, two different microenvironments closely interconnected.

Here, we reconstituted 3D spaces mimicking cancer and immune systems suitable to investigate the physical- and biochemical-driven interactions among these cell components. Specifically, we monitored the behaviour of IFN-DCs toward CRC cells, untreated or exposed to the innovative antitumor combined treatment with R and I (RI). We found that IFN-DCs moved through 3D immune and tumor spaces with a driven trajectory toward RI-treated cancer cells rather than untreated (NT) counterpart. Noteworthy, during dynamic migration, largely guided by the CXCR4/CCL12 axis, IFN-DCs were found to modify their motion in order to interact with drug-treated cancer cells and then to take up tumor Ags.

## Results

### A 3D microfluidic platform enables the evaluation of dynamic IFN-DC migration toward SW620 cancer cells exposed to combined treatment with IFN-α and romidepsin

**Figure 1:**
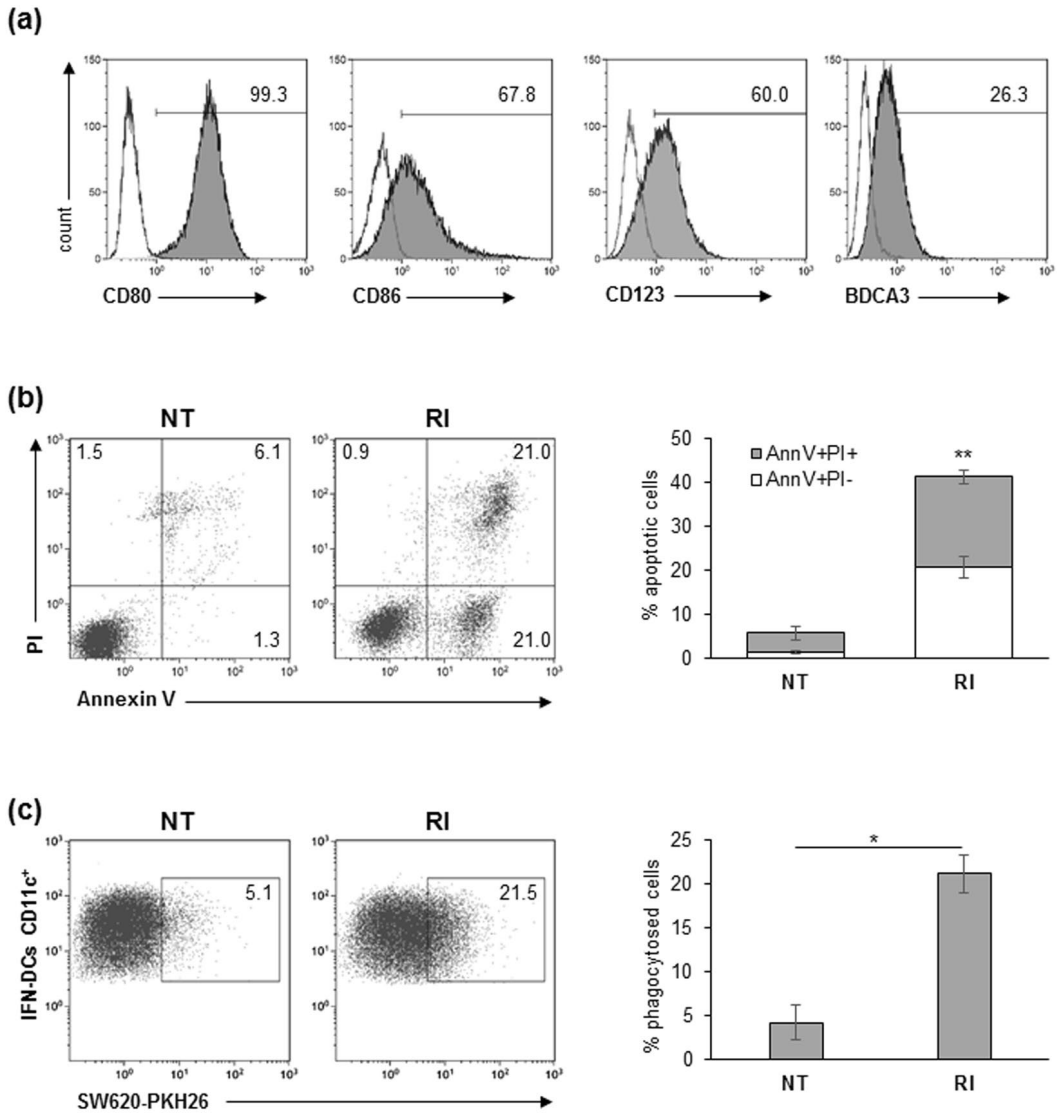
IFN-DCs efficiently phagocytose RI-treated SW620 cells. (**a**) Human monocyte-derived DCs generated in the presence of both IFN-α2b and GM-CSF were analysed by flow cytometry for the expression of maturation and differentiation markers. Percentages of positive cells (filled histograms) are shown; empty histograms represent isotype controls. Data are representative of three independent experiments. (**b**) Analysis of apoptosis of SW620 cells induced by 48 h-treatment with the combination of romidepsin and IFN-α2b (RI), as compared to untreated (NT) cancer cells. In the flow plots, early apoptotic cells (Annexin V^+^/PI^−^), late apoptotic cells (Annexin V^+^/PI^+^), necrotic cells (Annexin V^−^/PI^+^) and vital cells (Annexin V^−^/PI^−^) can be distinguished. One representative experiment out of four independent experiments is shown (left panel). In the right panel, percentages ± s.d. of AnnV^+^/PI^−^ and AnnV^+^/PI^+^ cells are shown (N = 4). (**c**) Flow cytometry analysis of phagocytosis of NT or RI-treated PKH26-labeled SW620 cells by IFN-DCs after a 4 h co-culture. IFN-DCs were stained with anti-CD11c mAb. One representative experiment (left panel) and the mean percentage ± s.d. of phagocytosed cells (right panel) are shown (N = 3).

**Figure 2:**
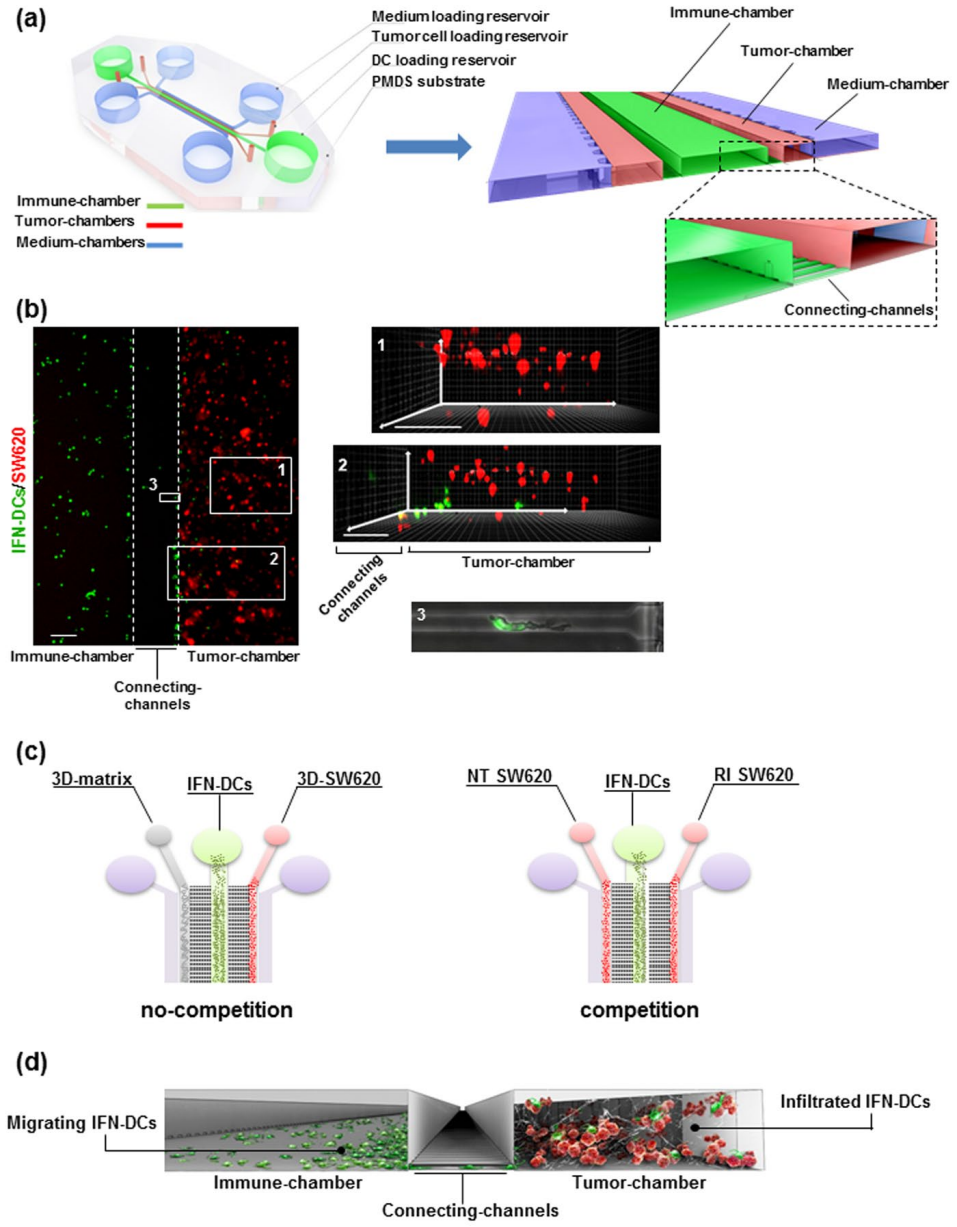
Design of the microfluidic device reconstructing the 3D immune-cancer spaces to track DC-cancer cells dialogue. (**a**) Schematic model of the 3D microfluidic device used for real time monitoring of DC migration toward cancer cells and their interactions. The device is composed of a 1.2 mm-wide central immune-chamber, with rounded loading reservoirs at both ends for DC loading, connected through a network of narrow microgrooves (connecting-channels) (10 × 12 × 200 µm, H × W × L) to two side tumor-chambers (150 × 500 × 1000 µm) end-closed with rounded loading reservoirs and designed to recreate 3D tumor spaces. Two external medium chambers ensure the optimal gas and nutrient exchange to the cell cultures. An enlarged 3D section of the device is shown. The boxed area represents a magnification of the connecting-channels. (**b**) Fluorescence image of the immune-tumor area of the device analysed in microfluidic experiments (left panel) showing the immune-chamber filled with PKH67 green-stained DCs, the connecting-channels and the tumor-chamber loaded with type I collagen embedded-PKH26 red-stained cancer cells. Images were acquired with an inverted Olympus IX73 microscope using a Plan Achromat 4×/0.10NA objective and processed with ImageJ software. Scale bar 100 μm. The inset photographs of the 3D projections represent: (1) a 3D rendering of a confocal image of SW620 cancer cells in the reconstructed 3D-matrix tumor space. Scale bar, 100 μm; (2) a 3D rendering of a confocal image of the connecting-channels entering the 3D-matrix tumor space showing IFN-DCs moving toward cancer cells; scale bar, 100 μm. Image stacks (100 images of 1 μm Z-step size) were acquired using a spinning disk microscope with a 20×/0.45NA objective and processed with Imaris software; (3) brightfield and green fluorescence merged image (20×) of a representative connecting-channel crossed by one IFN-DC actively moving toward the 3D-matrix tumor space. The image was processed with ImageJ software. (**c**) Schematic representation of isometric views of microfluidic devices showing the two experimental configurations used: no-competition and competition settings. (**d**) A 3D graphic representation of a section of the microfluidic device showing the interconnection between immune and tumor spaces and reproducing the motion of migrating DCs from the immune-chamber, crossing connecting-channels, toward the tumor-chamber, where cancer cells are targeted by infiltrated DCs.

The success of IFN-DC-based immunotherapy relies on the ability of these cells to accomplish Ag uptake by phagocytosis of dying cells in the TME and then to migrate to lymph nodes where they induce T cell-mediated antitumor responses^15, 30^. To investigate the interactions between IFN-DCs and SW620 CRC cells, we built a microfluidic platform suitable to recreate interconnected 3D spaces. To this end, we first generated primary IFN-DCs by culturing peripheral monocytes from healthy donors for 3 days with I and GM-CSF. In addition to the high expression of the co-stimulatory molecules CD80 and CD86, IFN-DCs were found to display the plasmacytoid CD123 marker as well as the type 2 myeloid DC marker BDCA3, which are involved in the cross-presentation of Ags derived from pathogens and dead cells^31, 32^ (Fig. 1a). Based on our previous data showing the ability of RI to promote DC recognition of cancer cells^8^, we then evaluated the phagocytic capacity of IFN-DCs toward SW620 cells pre-exposed to RI (RI SW620) for 48 h. We found that RI treatment markedly induced both early and late apoptosis in SW620 cells that in turn were phagocytosed at a higher rate by the IFN-DCs as compared to untreated cancer cells (NT SW620) (Fig. 1b,c). Therefore, in order to study the behaviour of IFN-DCs toward NT and RI SW620 we devised a novel strategy combining the design of a hydrogel-based microchannel platform with advanced microscopy as well as an unsupervised image processing algorithm. This approach allowed us to perform, on a single-cell basis, real time monitoring and analysis of DC migration as well as to evaluate the interactions occurring between IFN-DCs and SW620 cells. Figure 2a shows the microfluidic platform, containing a central immune culture compartment (immune-chamber) where floating IFN-DCs were uniformly distributed and showed non-adhesive motion, resembling the peripheral immune system. Two-side narrow chambers (tumor-chambers) were designed to establish 3D tumor microenvironments by embedding with a type I collagen matrix, where NT or drug-treated SW620 cells were seeded through two loading reservoirs, thus establishing 3D-NT and 3D-RI tumor spaces, respectively. On the basis of the already reported unique suitability of type I collagen for reproducing DC behaviour in the extracellular matrix^33^, it was used to build tumor-chambers re-creating proper biochemical and biophysical factors guiding DC migration and function. Each tumor-chamber, flanked on the external side by a parallel wide channel deputed to perfuse complete medium to the cultures, was connected to the immune-chamber via a set of short and narrow capillary migration channels (connecting-channels) (Fig. 2a,b, Supplementary Fig. 1). This geometry allowed to analyse DCs that move under confinement performing distinct processes such as migration, phagocytosis and cell-cell interactions, as it occurs *in vivo* during extravasation into peripheral tissues^34, 35^. For this purpose, two experimental configurations were used: (i) a no-competition condition, where the left tumor-chambers of two distinct platforms were functionalized with collagen without tumor cells (matrix) while collagen-embedded NT or RI-treated SW620 cells were cultured in the right tumor-chambers (Fig. 2c, left panel); (ii) a competition condition, where collagen-embedded NT SW620 and RI-treated SW620 cells (hereafter NT and RI) were placed in the left and in the right tumor-chambers, respectively, of the same platform (Fig. 2c, right panel). Such design provided the opportunity to re-create a microsystem characterized by interconnected tumor and immune compartments, yet retaining specific environmental properties. This model allowed to monitor the motility of IFN-DCs (migrating IFN-DCs) through the immune-chamber, connecting-channels, and tumor-chamber, in the presence of either non-competitive signals (no-competition) or biochemical stimuli (competition) and also to evaluate the overall recruitment of IFN-DCs into the 3D tumor space (infiltrated IFN-DCs) and their interactions with SW620 cells (Fig. 2d).

### Preferential IFN-DC migration toward the RI tumor environment

**Figure 3:**
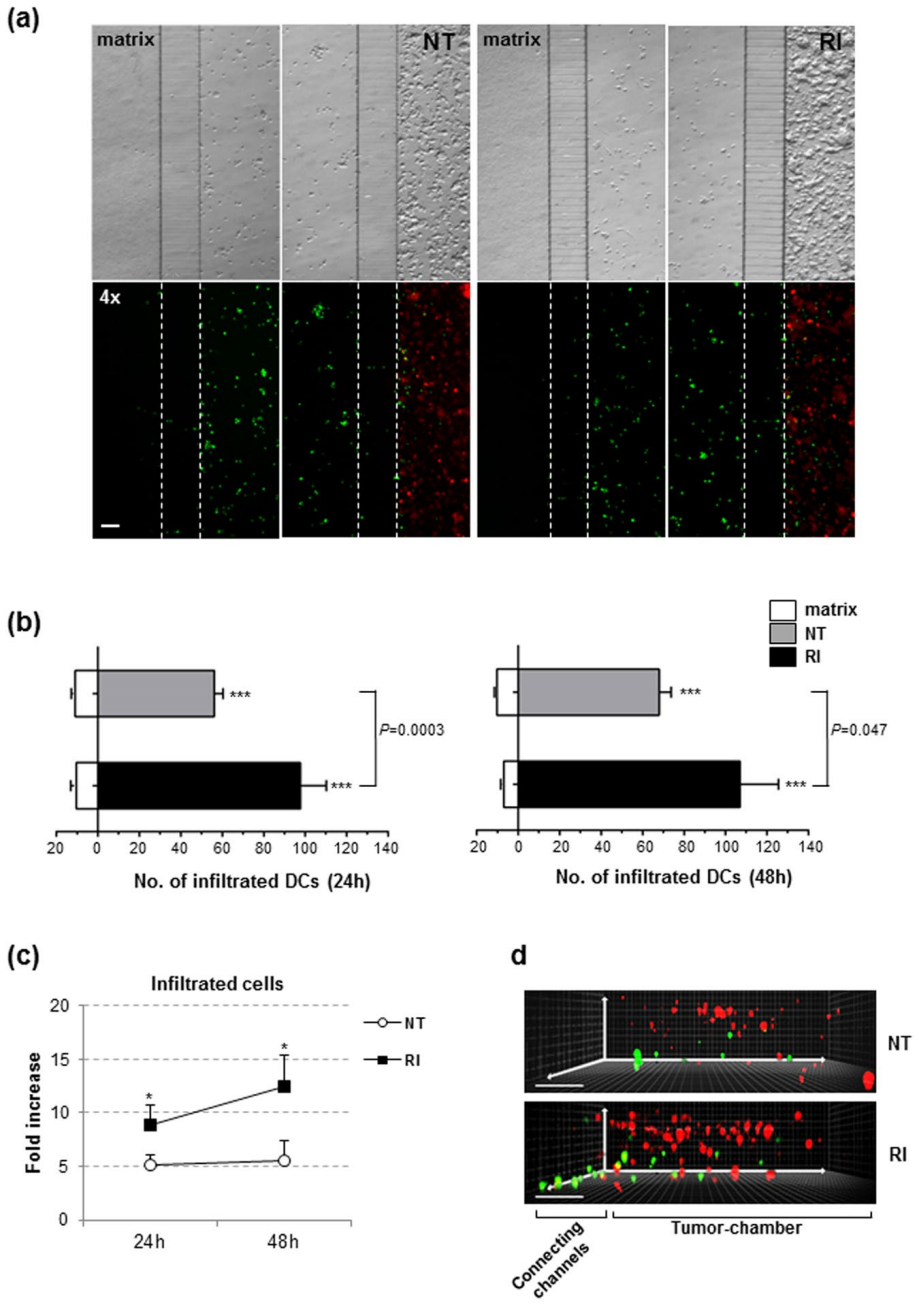
IFN-DCs migrate toward 3D tumor spaces. (**a**) Wide-field images of immune-tumor areas of microfluidic devices analysed in no-competition experiments. Brightfield images (top panels) and fluorescence images (bottom panels) show the preferential migration of IFN-DCs toward NT SW620 and RI SW620 spaces rather than an empty matrix. Images were acquired at 48 h from the start of migration using a Plan Achromat 4×/0.10NA objective and processed with ImageJ software. Scale bar, 100 μm. One out of three independent experiments is shown. (**b**) Distribution of infiltrated IFN-DCs into NT and RI spaces *versus* matrix, at 24 and 48 h. Means ± s.e.m. are shown. (N = 3 experiments; n = 6 and 5 for NT, 7 and 5 for RI, at 24 and 48 h, respectively. n = no. of fluorescence images analysed in each experiment). ***P ≤ 0.001, IFN-DCs migrating toward tumor chambers *versus* matrix. *P* = 0.5 and P = 0.047, infiltrated IFN-DCs into RI SW620 *versus* NT SW620 at 24 and 48 h, respectively (Student’s t-test). (**c**) Fold increase of infiltrating IFN-DCs into RI SW620 and NT SW620 spaces, with respect to the matrix. Means ± s.e.m. are shown (*P ≤ 0.05, Student’s t-test). (**d**) A 3D reconstruction of confocal image stacks (100 images of 1 μm Z-step size) of connecting-channels and tumor-chambers acquired using a 20×/0.45NA objective. PKH67 green-stained IFN-DCs actively migrate in the three spatial directions into the NT (upper panel) and RI (lower panel) spaces with an increased distribution into the 3D tumor space containing drug-treated PKH26 red-stained cancer cells. One representative image, for each image stack analysed, is shown. Three independent acquisitions were performed.

To explore the intrinsic propensity of IFN-DCs to migrate actively toward NT as well as RI-treated cancer cells, the microfluidic platform was used first under the no-competition condition. NT and RI tumor spaces were reconstituted into the right tumor-chamber of different microfluidic platforms with PKH26-stained cancer cells, depicted as red cells. Upon collagen polymerization, PKH67-stained IFN-DCs, visible as green-cells, were loaded and uniformly distributed into the immune-chamber. The capability of IFN-DCs to migrate toward the chambers filled with cancer cells rather than matrix alone was evaluated over a period of 24 to 48 h by wide-field imaging analysis of the entire microfluidic device, and the total number of IFN-DCs within the micro-channels and tumor-chambers was quantified. We found that, while IFN-DCs did not recognize the matrix, they actively moved from the immune-chamber toward both tumor-chambers containing NT and RI-treated SW620 cells through the connecting-channels (Fig. 3a). Consistently, the number of infiltrated IFN-DCs into the 3D-tumor spaces was significantly higher than that infiltrated into the matrix. Notably, at both time points we found that the number of IFN-DCs infiltrated into the RI space was significantly higher compared to that into the NT space (Fig. 3b), and a remarkable fold increase of infiltrated IFN-DCs, with respect to matrix, was observed in the RI space (Fig. 3c). Of interest, IFN-DCs in both cases were able to migrate upward and migrate deep within the 3D-tumor space (Fig. 3d).

**Figure 4:**
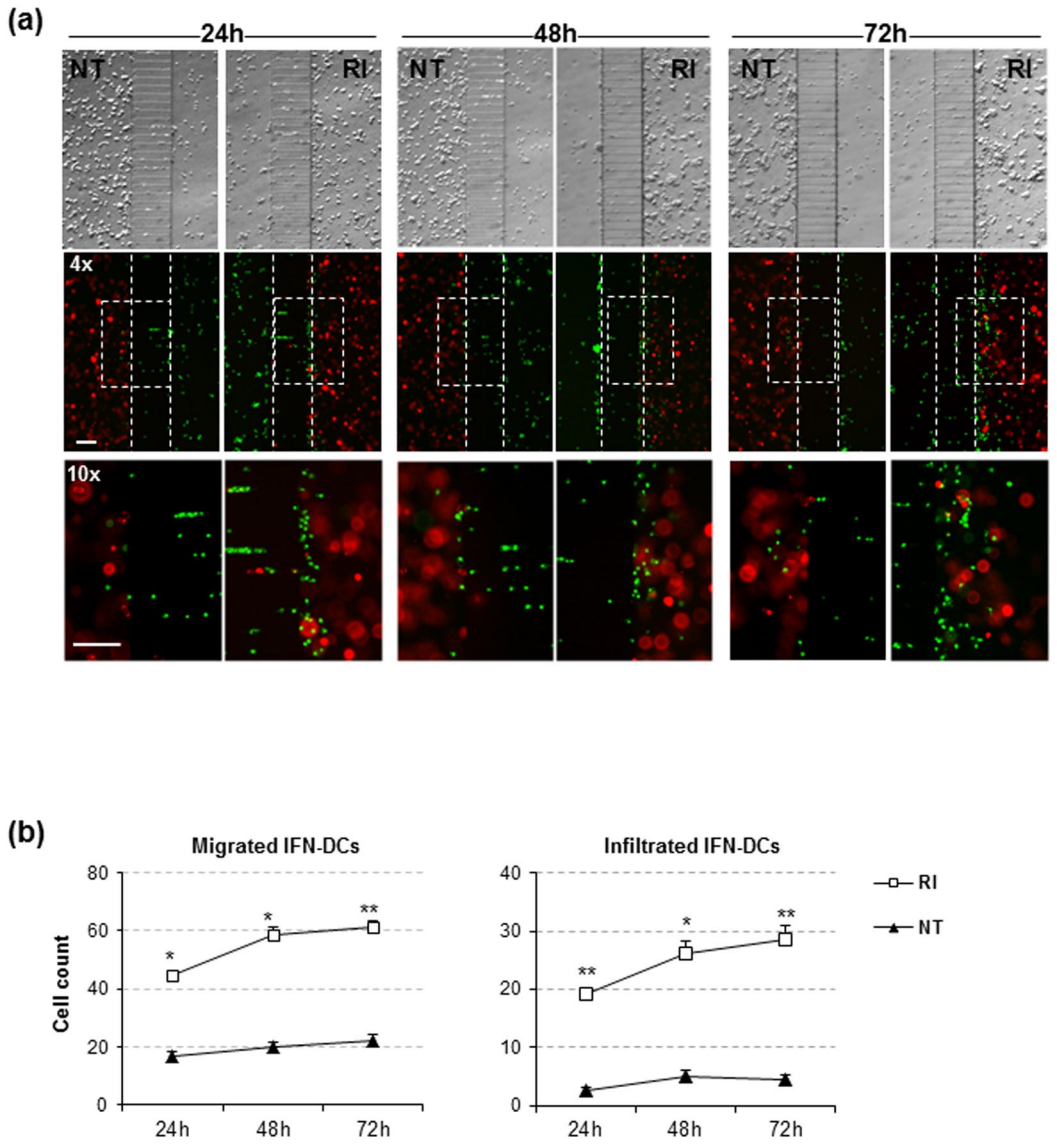
IFN-DCs preferentially migrate toward RI-treated SW620 cells. (**a**) Images of immune-tumor areas of the microfluidic devices analysed in competition experiments. Brightfield images (top panels) and fluorescence images (middle panels) acquired using a Plan Achromat 4×/0.10NA objective show the preferential migration of PKH67 green-stained IFN-DCs toward RI *versus* NT spaces containing PKH26 red-stained cancer cells at 24, 48 and 72 h observation time points (Scale bar, 100 μm). Bottom panels represent the magnification of boxed areas in the middle panels, acquired by 10×/0.25NA objective (Scale bar, 100 μm). One representative experiment out of three is shown. (**b**) Kinetics of migrating IFN-DCs, represented by cells counted in the analysed connecting-channel and tumor-chamber areas, and infiltrating IFN-DCs, represented by cells counted in the analysed tumor-chamber area, showing the massive recruitment of these cells by drug-treated SW620 cells. Images acquired with 10× magnification were quantified. Means ± s.e.m. are shown (*P ≤ 0.05, **≤0.01, Student’s t-test) (N = 3 experiments; n = 32, 30 and 34 for NT; n = 29, 26 and 29 for RI, at 24, 48 and 72 h, respectively. n = no. of fluorescent images analysed).

**Figure 5:**
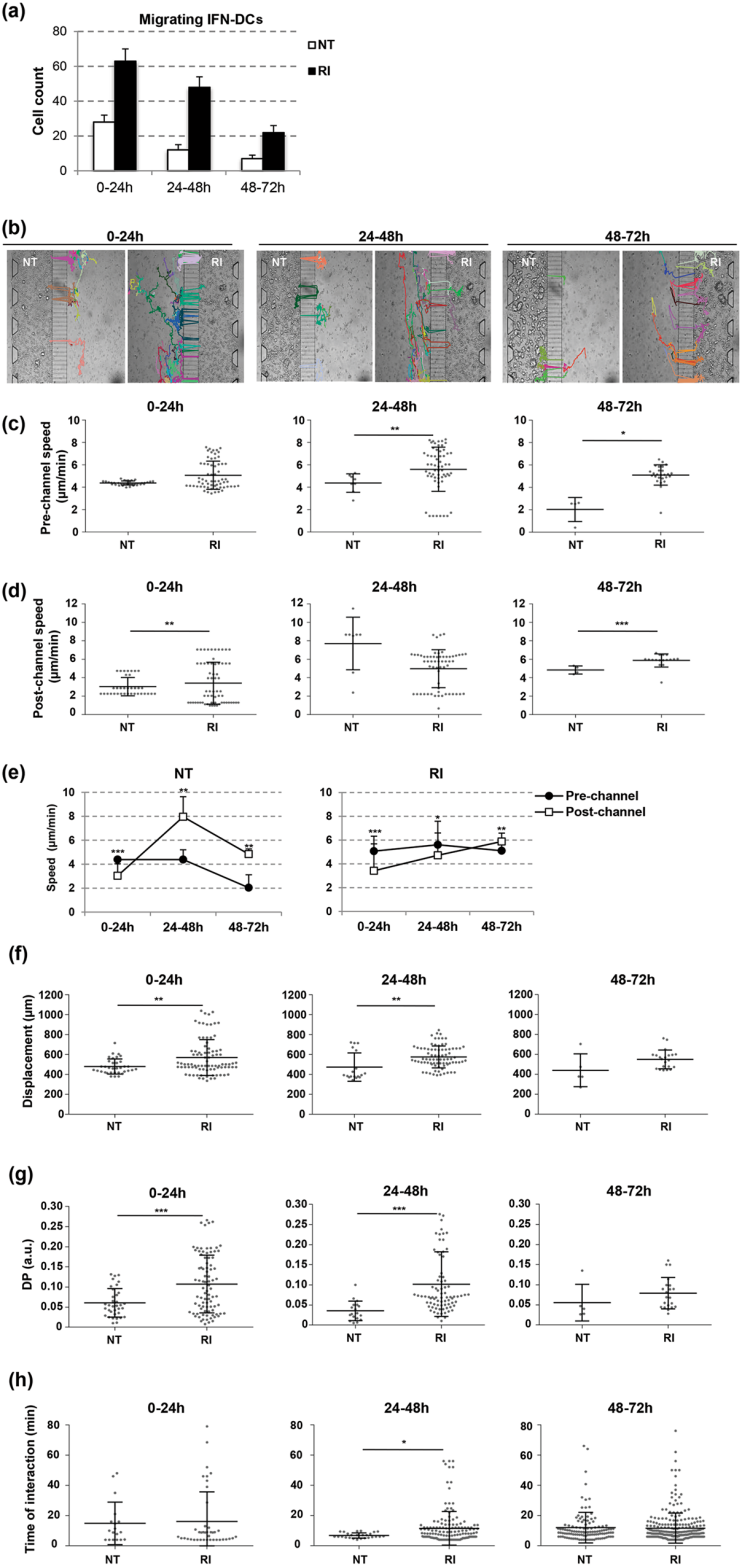
Analysis of IFN-DCs and RI-treated SW620 cells interaction. (**a**) Quantification of migrating IFN-DCs crossing connecting-channels towards NT or RI-treated SW620 cells by the revised unsupervised algorithm for cell tracking analysis of 0–72 h time-lapse recording. Images were acquired every 2 min by a Juli Smart microscope (4× magnification). Means ± s.d. are shown. Three independent experiments were analysed. (**b**) Migratory patterns of IFN-DCs toward NT or RI-treated SW620 cells were analysed in 0–72 h time-lapse experiments with cell tracking analysis. Paths of each IFN-DC crossing the connecting-channels from the immune-chamber in the direction of either NT or RI tumor-chambers are shown. Representative photos taken from three independent video recordings are shown. (**c**,**d**) Scatter plots of the migratory speed of IFN-DCs in the immune-chamber (pre-channel speed) and in the tumor-chambers (post-channel speed) both toward NT and RI-treated SW620 cells. Each data point represents the average velocity of one cell over the time periods of 0–24, 24–48 or 48–72 h. Horizontal bars indicate mean ± s.d. (**e**) Pre- and post-channel migratory speed of IFN-DCs toward and into the NT and RI-treated SW620 cell space over 0–72 h period. (**f**) Total horizontal path displacements of IFN-DCs. Each data point represents the average displacement of one cell over the time periods of 0–24, 24–48 or 48–72 h. Horizontal bars correspond to mean ± s.d. (**g**) IFN-DC migratory persistence recorded in time-lapse experiments. The DP of IFN-DCs is significantly higher toward RI than NT SW620 cells for up to 48 h. Each data point represents directional persistence of one cell over the time periods of 0–24, 24–48 or 48–72 h. Horizontal bars correspond to mean ± s.d. (**h**) Time of interaction between one IFN-DC and one SW620 cell calculated into the NT and RI tumor-chambers over the time periods of 0–24, 24–48 or 48–72 h. The value was based on a defined circular region of interaction around each cancer cell (see Supplementary Information for details). Horizontal bars represent mean ± s.d. (*P ≤ 0.05, **≤0.01, ***≤0.001, Student’s t-test).

To further characterize the propensity of IFN-DCs to migrate preferentially toward drug-treated cancer cells, we performed experiments under the competition condition. The behaviour of IFN-DCs was evaluated over three time periods: 0*–*24 h, 24*–*48 h and 48–72 h. Wide-field imaging analysis showed that over the entire follow-up period IFN-DCs moved preferentially toward the RI-treated cancer cells, resulting in a constant higher number of infiltrating IFN-DCs into the RI tumor space as compared to that into the NT tumor space (Fig. 4a,b and Supplementary Fig. 2). Next, the 3D dynamics of the IFN-DC migration toward the cancer cells under the competition condition were captured by time-lapse recordings during a 0–72 h period (Supplementary Movies 1 and 2). Taking advantage of an adapted unsupervised cell tracking analysis algorithm, a quantitative analysis of IFN-DC migration was performed by evaluating the following parameters: (i) number of migrating IFN-DCs accomplishing active motion across the immune-chamber, connecting-channels and tumor-chamber; (ii) velocity of IFN-DCs in the immune-chamber before entering the connecting-channels (Pre-channels speed); (iii) velocity of IFN-DCs in the tumor-chamber after passing through the connecting-channels (Post-channels speed); (iv) IFN-DC displacement; (v) directional persistence (DP) of IFN-DC migration; and (vi) average time of interaction between IFN-DCs and SW620 cells at the single-cell level. First, we defined the environments (background elimination step) and cells (segmentation step) in each frame of the three independent time-lapse experiments (Supplementary Fig. 3a,b and Supplementary Methods). By choosing the same areas of the microfluidic platforms, we identified trajectories of migrating IFN-DCs with single-cell resolution and traced the single cell movement from the immune-chamber to the tumor-chamber over a 0–72 h period (Supplementary Table 1, Supplementary Fig. 4a). Software results were validated by manual ImageJ analysis (Supplementary Fig. 4b). We found much greater migration of IFN-DCs toward RI relative to that toward NT counterpart overtime, with the tendency for the RI-induced migration to reduce gradually (Fig. 5a,b). Importantly, IFN-DCs localized in the immune-chamber next to the RI space, where they can sense tumor-specific stimuli, moved with a higher constant speed as compared with those cells positioned in the corresponding area next to the NT space (0–24 h: 5.1 μm/min vs 4.4 μm/min; 24–48 h: 5.6 μm/min vs 4.4 μm/min; 48–72 h: 5.1 μm/min vs 2.0 μm/min) (Fig. 5c and Supplementary Table 1). Of note, real time imaging revealed that IFN-DCs crowded the entrance and showed promiscuous crossing through the connecting–channels next to the RI space, in contrast the IFN-DCs moved toward the NT space by random walking (Supplementary Movie 3). Of interest, once in the RI space, IFN-DCs significantly reduced their intrinsic movement during a 0–48 h period, in contrast to the cells that migrated into the NT space that exhibited a slight speed reduction up to 24 h but increased the migratory speed over 48–72 h (0–24 h: 3.4 μm/min vs 3.0 μm/min; 24–48 h: 4.98 μm/min vs 7.7 μm/min; 48–72 h: 5.8 μm/min vs 4.9 μm/min) (Fig. 5d,e). These findings suggest that IFN-DCs are endowed with high capability to adapt their migration in response to external signals^36, 37^. Moreover, migrating IFN-DCs were found to move in remarkably higher number toward RI, characterized by higher DP, relative to the migration toward NT overtime (0–24 h: 571 μm vs 480 μm, 24–48 h: 576 μm vs 474 μm, 48–72 h: 550 μm vs 441 μm) (Fig. 5f,g). Next, we characterized IFN-DC and cancer cell interactions and found that over 24–48 h single IFN-DCs exhibited a longer average interaction time with single cancer cells in RI relative to that in NT space (0–24 h: 16.1 min vs 14.9 min, 24–48 h: 11.5 min vs 8.7 min, 48–72 h: 11.6 min vs 12 min) (Fig. 5h).

### The CXCR4/CCL12 axis guides IFN-DC movement toward the RI space to capture apoptotic cancer cells

**Figure 6:**
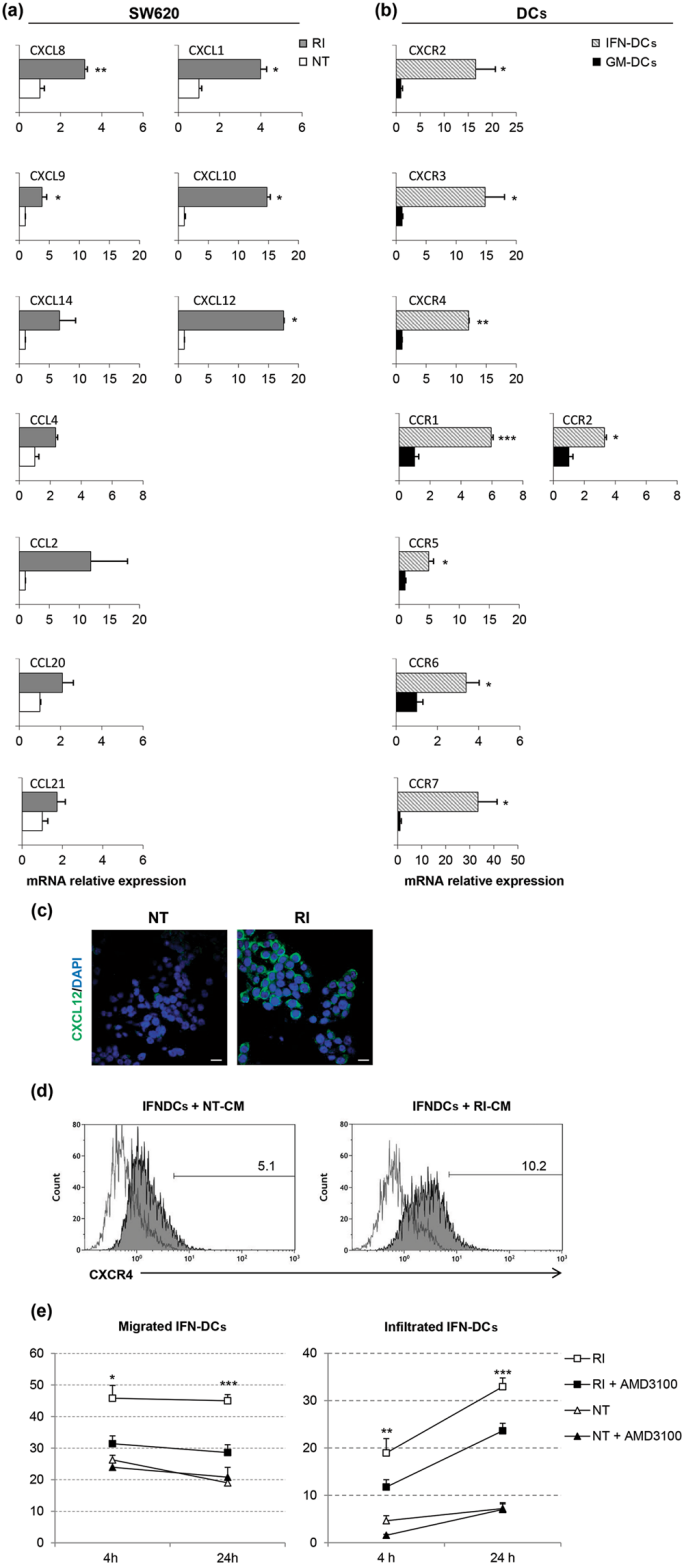
The CXCR4/CXCL12 axis drives the migration of IFN-DCs toward RI-treated SW620 cells. Analysis of the expression of CC-/CXC-chemokines in SW620 cells (**a**) and of relative chemokine receptors in IFN-DCs (**b**) by qPCR. Expression values in the RI-treated SW620 cells and in the IFN-DCs are relative to the NT SW620 cells and to the GM-DCs, respectively (means ± s.e.m., *P ≤ 0.05, **≤0.01, ***≤0.001). (**c**) Immunofluorescence analysis of CXCL12 expression on both NT and RI-treated SW620. Images were collected with the same acquisition parameters. Scale bar, 20 µm. (**d**) Analysis of CXCR4 expression by flow cytometry on IFN-DCs after exposure to CM from 48 and 72 h NT and RI-treated SW620 cells (N = 3). (**e**) IFN-DC migration toward RI-treated SW620 cells is significantly inhibited by the CXCR4 inhibitor, AMD3100. Migration in the presence or absence of AMD3100 were performed in microfluidic devices, and the acquired images were quantified at 4 and 24 h (magnification 4×). Means ± s.e.m. are shown (*P ≤ 0.05, **≤0.01, ***≤0.001, Student’s t-test) (N = 3 experiments; a means of 10 images for group were analysed).

**Figure 7:**
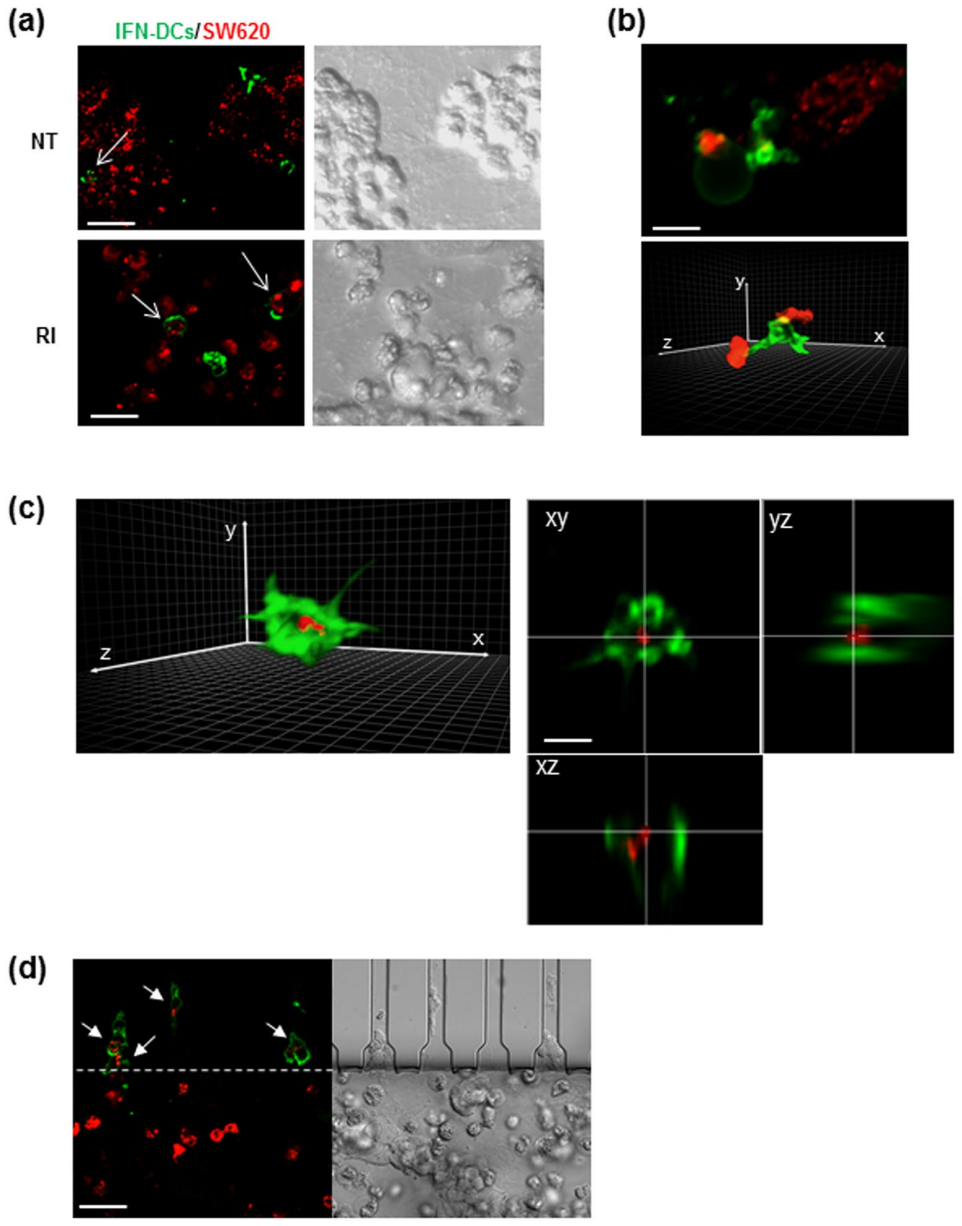
IFN-DCs efficiently phagocytose RI-treated SW620 cells. (**a**) Fluorescence images (left panels) of HLA-DR-stained IFN-DCs (green) performing phagocytosis of PKH26-stained cancer cells (red) into NT or RI spaces, as evaluated by CLSM after 48 h from the beginning of migration in the microfluidic device under competition experimental conditions. The right panels show DIC (Differential Interference Contrast) images of the same fields. Images acquired using a UPLANSAPO 20×/0.75NA objective are shown. Zoom 3; scale bar, 15 μm. To avoid crosstalk between different fluorophores, sequential acquisition was performed. Data are representative of three independent experiments. (**b**) A 3D rendering of image stacks (16 slices of 0.5 μm Z-step size) acquired within the RI tumor space. Zoom 6; scale bar, 5 μm. One IFN-DC extending trans-cellular dendrites toward SW620 cells is depicted in green (top panel) and one IFN-DC establishing contact with two different SW620 cells is depicted in green (bottom panel). (**c**) A 3D rendering of one IFN-DC (green) engulfing one SW620 cell (red) from the image stacks (16 slices of 0.5 μm Z-step size) acquired within the RI tumor space (left panel). Orthogonal sections of the phagocytic event depicted in the right panel are shown (left panels). Scale bar 5 μm. In (**b** and **c)**, a 3D rendering was edited by Adobe Photoshop CC software preserving the original parameters of image acquisition (**d**) Fluorescence image showing IFN-DCs (green) crossing connecting-channels facing RI space upon phagocytosis of PKH26-stained SW620 cells (red) as evaluated by CLSM after 48 h from the beginning of migration in the microfluidic device, under competition experimental conditions. The right panel shows a DIC image of the same field. Images are acquired as in (**a**). Scale bar, 15 μm. Representative images of two independent experiments are shown.

DC migration within the TME and toward lymph nodes is driven by a fine-tuned regulation of chemokine and chemokine-receptor expression^38^. Indeed, the IFN-DC maturation stage and functional activities have been reported to be regulated by responsiveness to chemokines^14^. In this paper, we further investigated the role of chemokines expressed by RI-treated SW620 cells in regulating the movement of IFN-DCs. RI-treated SW620 cells were found to express higher levels of CCL2, CCL4, CCL20, CCL21 as well as CXCL1, CXCL8, CXCL9, CXCL10, CXCL12 and CXCL14 mRNA relative to their NT counterpart (Fig. 6a). Accordingly, IFN-DCs exhibited elevated expression of corresponding chemokine receptor transcripts, namely CCR1, CCR2, CCR5, CCR6 and CCR7 as well as CXCR2, CXCR3, and CXCR4 (Fig. 6b). Next, a key role of the CXCR4/CXCL12 axis in the guided movement of IFN-DCs toward the RI space was defined. In fact, after SW620 cells were treated with RI for 48 h, they produce CXCL12 in significant amounts (Fig. 6c). Moreover, IFN-DCs exposed to conditioned medium (CM) from RI-treated SW620, selectively increased CXCR4 expression on the membrane compared to that of cells exposed to CM from NT cancer cells (Fig. 6d). Therefore, we performed migration experiments in the microfluidic device in the presence of the CXCR4 inhibitor AMD3100. We found that while IFN-DC migration toward NT was not affected by CXCR4 inhibition, the IFN-DC migration toward the RI space was significantly reduced by AMD3100 already by 4 h, persisting for up to 24 h (Fig. 6e and Supplementary Fig. 5a). The inhibition of CXCR4/CXCL12-driven IFN-DC migration by AMD3100 was confirmed in a Transwell migration assay (Supplementary Fig. 5b). It was previously shown that DC engulfment of damaged cancer cells following their scanning of the TME is pivotal for inducing antitumor immunity^35^. Consistently, we observed that IFN-DCs were able to more efficiently phagocytose RI SW620 cells compared to NT counterpart (Fig. 7a). IFN-DCs could migrate in three directions within the RI space, confirming the add value of our model in investigating the guided movement of DCs (Supplementary Movie 4). In fact, we traced the migration of a single IFN-DC toward a single cancer cell in the RI space and thus highlighted the events of phagocytosis (Supplementary Movie 5). In this process, IFN-DC moved slowly (Fig 5d,e), extended trans-cellular dendrites to establish contact with cancer cells (Fig. 7b), and finally phagocytosed the cancer cell (Fig. 7c). We also found some tumor Ag-loaded IFN-DCs migrating back into the connecting-channels (Fig. 7d), suggesting the occurrence of a second moving phase after phagocytosis resembling DC migration in draining lymph nodes after capture of tumor antigens^39^.

## Discussion

In this paper, we exploited a novel microfluidic platform recreating interconnected 3D immune and tumor systems, to study the behaviour of IFN-DCs toward cancer cells upon treatment with two complementary antitumor agents, the epigenetic drug romidepsin and the immunomodulator IFN-α^8^. We identified the processes undertaken by IFN-DCs for approaching dying cancer cells for Ag uptake. In particular, the following events were monitored and evaluated in real time: (i) IFN-DC scanning of environmental signals and adjustment of migratory capacity toward RI-treated cancer cells; (ii) modulation of movement parameters by IFN-DCs in performing guided migration in a confined space; (iii) establishment of IFN-DC-cancer cell interactions leading to Ag uptake.

DC-based immunotherapy is a promising tool for cancer treatment currently under intense investigation for its unique potential to initiate and direct Ag-specific T cell immune responses^40^. Furthermore, improved clinical protocols suggest that the combination of DC-based therapies with other agents may enhance the potency of the antitumor effects^41^. In this landscape, IFN-DCs are promising candidates to be combined with agents that promote cancer cell death and favour Ag uptake^42^. Here, we tested this idea and found that a high rate of CRC cell apoptosis following RI treatment resulted in a significant increase of phagocytosis by IFN-DCs. The choice to treat cells with RI in combination relies on the complementary antitumor properties of these drugs. While the direct and immune-mediated antitumor properties of IFN have recently renewed interest in the use of this cytokine in new therapeutic protocols^43^, HDACi have been reported to induce both cancer cell death and immunomodulatory effects^44^. Accordingly, we found that the RI combination was able to exert direct anti-tumor effects and to drive DC functions against cancer cells. Importantly, although these events represent pivotal markers of the efficacy of immunotherapy their detection remains a true challenge^45^. Therefore, in the attempt to overcome this limitation, we employed a microfluidic approach for dissecting the intimate dialogue between IFN-DCs and RI-treated SW620 cells. In the past few years, the rapid technological advances have led to the development of new platforms for monitoring in real time the events linked to cancer development^46^. In order to examine the dialogue between cancer and immune cells, we previously employed a simplified microfluidic device. Spleen cells from mice knocked out for IRF-8, compartmentalized across a network of microchannels, approached cancer cells only by random movements, whereas single immunocompetent splenocytes accomplished drifted random migration leading to a highly coordinated response^27, 28^. Importantly, these data agreed with *in vivo* functional results^47^. Herein, the new design of the microfluidic platform allowed building of immune-tumor 3D spaces similar to the physiological environment characterized by simultaneous compartmentalization as well as by interconnections of the tumor and immune systems. Both the physical device architecture and the type of 3D matrix, that regulate the phenotype and functions of cells^35, 48^, were taken into consideration for reproducing spaces mimicking *in vivo* environments where DCs and cancer cells migrate and interact. First, immune-chamber dimensions recreated a spatial confinement, mimicking vessels and tissue interstitial areas, in which DCs, under non-specific adhesive conditions, were able to tune guided migration upon sensing factors released by tumor cells^49^. In addition, the geometry of the connecting-channels allowed DCs to migrate actively through very narrow constrictions to the same extent as when they cross endothelial barriers^50^. On the other hand, the microarchitecture, porosity and stiffness of type I collagen conferred tumor spaces with the ability to release biochemical signals from dying cells to guide and adapt DC movements^51^. Therefore, our microfluidic platform was able to recapitulate physical and biochemical environmental cues driving the interactions between DCs and tumor cells, closely resembling *in vivo* conditions in a time- and spatial-dependent manner. Complementary events, undetectable on 2D flat surfaces used in traditional assays^49^, such as IFN-DC migration and interaction with single cancer cell, were linked in real time using high-resolution time-lapse imaging. Moreover, different parameters characterizing IFN-DC behaviour toward NT and RI-treated cancer cells were evaluated with an automated data analysis system using a revised image analysis algorithm thus overcoming for the first time one of the most important challenges of microfluidic studies, i.e. time-consuming manual data analysis^52^. Specifically, IFN-DCs when sensing stimuli exclusively from either NT or RI-treated SW620 cells displayed a propensity to be recruited into the tumor mass but not in the competing matrix space. In this scenario, IFN-DC migration toward the RI space was significantly higher than that of cells recruited toward NT space. However, the elevated attractant potential of the molecules released by RI-treated dying cells toward IFN-DCs was clearly evident under competition conditions with the massive recruitment of these cells into RI tumor space relative to the NT counterpart. In this case, migrating IFN-DCs adjusted their migratory properties with respect to their functional activity^49^. First, in the immune-chamber they acquired higher pre-channel speed generating crowding in entrance at connecting-channels where they acquired an “amoeboid-like” locomotion mode, with dendritic protrusions at the cell front and contractions at the cell rear, propelling the nucleus through narrow spaces thus allowing persistent cell migration^53^. Once in the RI space, IFN-DCs significantly reduced their velocity, in agreement with Ag internalization along with a concomitant decrease in migration upon maturation stimuli^54^. IFN-DCs migrating toward the RI tumor space had longer displacements with high DP, in contrast to the migration toward the NT space. In line with the evidence that chemokines may drive DC behaviour^38^, biochemical signals released in the RI tumor space drove IFN-DC migration. Enhanced transcript expression of several chemokines by RI-treated tumor cells was observed along with high levels of the corresponding chemokine receptors on the IFN-DCs. Such chemokines drive DC recruitment into inflamed sites^55^. Of note, elevated CXCL12 production by RI-treated dying cells agreed with the increased expression of CXCR4 on IFN-DCs, a receptor known to guide mature DC motility^56^, thus contributing to IFN-DC migration toward RI tumor space. The key role of the CXCR4/CCL12 axis in driving IFN-DC chemotaxis toward the RI space was further confirmed by the functional inhibition with the specific CXCR4 inhibitor. This finding is consistent with the report showing that CXCR4 as well as the cooperation between both inducible CXCR3 ligands and constitutive CXCL12 regulate DC trafficking to peripheral sites^57^. On the contrary, the high CCR7 expression on IFN-DCs did not associate with the induction of CCL21 transcripts on cancer cells, suggesting a minor role of the CCR7/CCL21 axis for the movement and function of IFN-DCs under these conditions.

The further analysis of DC-cancer cell interactions revealed a high competence of IFN-DCs to take up Ags by exploring the RI tumor space. In fact, the time of interaction between one IFN-DC and one SW620 cell was higher in the RI space than that in the NT space over 0–48 h period, whereas it decreased along with the augmented migration velocity during the subsequent 48–72 h period. These data support the superior competence of IFN-DCs in patrolling drug-treated space with alternating migration phases characterized by pausing times for endocytosis^12^. IFN-DCs also established communications with cancer cells throughout trans-cellular dendrites when the velocity was reduced, suggesting the capability of these cells to modulate multiple functions. Finally, we also report that Ag-loaded IFN-DCs may make a directional choice toward the connecting-channels pointing out the potential of our platform for studying DC and T lymphocyte interactions, after Ag uptake^58^.

In summary, the microfluidic model developed in this study establishes the proof of concept for real time tracking of immune-tumor cell interactions and validates the efficacy of combination strategies in potentiating DC-based immunotherapy. Beyond this unquestionable advantage, this platform offers a valid and manageable alternative to studies in animals for evaluating the efficacy of novel immunotherapeutic treatments while its further implementation may lead to the development of suitable assays for pre-clinical testing.

## Methods

### Cell Culture

IFN-DCs were generated using a standard protocol previously described^59^. Briefly, CD14^+^ monocytes were isolated from human peripheral blood mononuclear cells (PBMCs) and cultured with both 500 U/ml GM-CSF (PeproTech, NJ) and 10^4^ IU/ml IFN-α2b (Intron A, Merck Sharp and Dohme, Kenilworth, NJ) for 3 days. Then, the floating cells were collected and used for experiments. CD14^+^ monocytes were also cultured for 3 days with GM-CSF alone, obtaining GM-DCs that were used as control in the quantitative real-time PCR (qPCR) experiments. For 3D microfluidic migration experiments, IFN-DCs were stained with PKH-67 Fluorescent Cell Linker (Sigma-Aldrich), according to the manufacturer’s instructions. Low passage-number SW620 CRC cells were maintained in RPMI-1640 (Lonza, Verviers, Belgium) with 10% FCS (EuroClone, West York, UK). Cells were seeded at 3.5 × 10^4^/cm^2^ and then 24 hour later were treated with or without 2 nM romidepsin® and 10^4^ IU/ml IFN-α2b (I) in combination (RI) for 48 h. For 3D microfluidic migration experiments, SW620 cells were stained with PKH-26 Fluorescent Cell Linker (Sigma-Aldrich), according to the manufacturer’s instructions.

### Flow cytometry

For the detection of drug-induced cell death, NT or RI-treated SW620 cells were stained with Annexin V-FITC and propidium iodide (PI), according to the manufacturer’s instructions. Data were acquired using FACS Calibur (Becton Dickinson, BD) and analysed by either WINMDI or Kaluza softwares (Beckman Coulter). For the immunophenotypic analysis, IFN-DCs were stained with the following monoclonal antibodies (mAbs): anti-CD86 (APC, clone 2331), anti-CD80 (APC-H7, clone L307.4), anti-CD123 (Brilliant Violet, clone 9F5) and anti-BDCA3 (PE clone 1A4), all from Becton Dickinson. Data were acquired by a Gallios flow cytometer (Beckman Coulter) and analysed by Kaluza software. For phagocytosis quantification, PKH-26-labelled SW620 cells were treated with or without RI. After 48 h, dying floating cells were harvested and incubated with IFN-DCs at a 1:2 ratio in conic 15 ml tissue culture tubes at 37 °C for 4 h. Then, all cells collected from the co-culture were stained with anti-CD11c mAb (APC, clone B-ly6, BD) followed by flow cytometry. The uptake of apoptotic cells by IFN-DCs was determined by calculating the percentage of double-positive cells (CD11c-APC^+^/Apo-PKH26^+^). All gates were set on SW620-IFN-DC co-cultures performed at 4 °C, keeping unchanged all the other experimental conditions. The analysis was performed with Kaluza software (Beckman Coulter). To analyse CXCR4 expression, IFN-DCs were cultured at 2 × 10^6^/ml for 24 h with conditioned medium (CM) derived from either 48 h or 72 h cultures of NT or RI-treated SW620 cells. Stimulated or not stimulated IFN-DCs were collected and stained with anti-CXCR4-PE mAb (PE, clone 12G5, eBioscience) and with anti-CD11c-APC (BD). Sytox dye (Thermofisher Scientific, Waltham, MA USA) was used to gather the dead cells. CXCR4^+^/CD11c^+^ positive cells were quantified by a Gallios flow cytometer (Beckman Coulter) and were analysed by Kaluza software.

### Fabrication of the microfluidic devices

3D co-culture experiments were performed in an ad hoc-designed cell-culture microfluidic device fabricated at the CNR-IFN facility according to already published protocols^60^. PDMS replica and microscope glass slides were plasma oxidized (Oxford Plasma Lab 80 plus, RF Power: 20 W, Flux: 60 sccm, Pressure: 800 mtorr, Time: 30 s) and bonded together at 90 °C for 4 h on a hotplate. Selective hydrophilic activation of chamber walls by O_2_ plasma treatment was needed to guarantee the correct gel matrix positioning and the proper filling of connecting-channels before biological experiments. Microfluidic co-culture experiments were performed under static conditions, with passive diffusion of medium, oxygen and all molecular components.

### Cell loading and culture in the microfluidic platform

Devices were sterilized under UV light in a laminar flow hood for 15 min before loading cells. Rat-tail type I collagen (Corning, MA, USA) was prepared at 3 mg/ml, according to the manufacturer’s instructions, and the pH was adjusted to 7 with 1 N NaOH. Collagen was kept for 30 min on ice before adding 25 × 10^4^ PKH-26 labelled-NT or RI-treated SW620 cells. Then, 3.5 μl of cell/collagen mixtures were inoculated into the tumor-chambers. In some experiments, one of the tumor-chambers was filled with only collagen, without tumor cells. Gels were allowed to polymerize at 37 °C for 30 min in a tissue culture incubator (5% CO_2_). Devices were filled with complete medium and stabilized at 37 °C for 30 min. Then, 5 × 10^5^ PKH-67 labelled-IFN-DCs were inoculated, through the loading reservoir, into the immune-chamber, after removing the medium. All reservoirs were then filled with 100 μl medium. Migration experiments in the presence of CXCR4 inhibitor were performed by adding 20 µM AMD3100 (Sigma-Aldrich) to the IFN-DCs before loading into the devices. For time-lapse experiments with the Juli microscope, IFN-DCs and SW620 cells were loaded into the devices in the absence of fluorescent staining.

### Microscopy

Wide field live cell imaging was carried out with an inverted Olympus IX73 microscope equipped with a Lumencor Spectra X LED illumination using an Evolve Delta CCD camera (Photometrics) and MetaMorph V7.8.0 software (Molecular Devices). The Oko-lab incubation system was employed to control temperature, humidity and CO_2_ during acquisition. Fluorescence and brightfield images of the entire device were collected at 24, 48 and 72 h both with a Plan Achromat 4×/0.10NA and 10×/0.25NA objectives (Olympus), and processed to quantify DC migration by automatic cell counting using ImageJ software (NIH). 3D movies were made by acquiring wide field image stacks (100 images of 1 μm Z-step size) every 5 min for each wavelength using a UPLANFL long working distance 20×/0.45NA objective (Olympus). Images were processed with Imaris software v.8.1.2 (Bitplane). For 3D reconstruction confocal images were acquired using a X-light Nipkow spinning-disk head (Crest Optics). Images of 1 μm Z-step size were acquired for each wavelength (n = 100) and processed with Imaris software. Image stacks were edited by Adobe Photoshop CC Software (Adobe Systems) preserving the original parameters of image acquisition. To analyse CXCL12 expression, SW620 cells were treated with or without RI for 48 h. Cells were then fixed with 4% PFA for 15 min at 4 °C and stained with anti-CXCL12 mAb (R&D, clone 79018). Images were taken with a FV1000 confocal microscope (Olympus, Tokyo, Japan) using a (Olympus) planapo objective 60× oil A.N. 1,42. Excitation light was obtained by a Laser Dapi 408 nm for DAPI, and an Argon Ion Laser (488 nm) for FITC (Alexa 488). DAPI emission was recorded from 415 to 485 nm and FITC emission was recorded from 495 to 550 nm.

### Cell tracking analysis

IFN-DC migration toward and within the 3D tumor spaces was recorded using time-lapse imaging on a Juli Smart microscope placed in a CO_2_ incubator at 37 °C for 0–72 h. The unsupervised image analysis algorithm used for 2D cell tracking in a 3D environment is articulated into three main steps: (i) recognition of confined environment of interest in the microfluidic platform (background elimination); (ii) recognition of cells (segmentation); and (iii) construction of connections (linking)^61^. See Supplementary Methods for details. Once spaces and cells were identified in the immune-chamber, IFN-DC motion was analysed by evaluating the following parameters^62^: (i) number of migrating IFN-DCs performing active migration from the immune-chamber to the tumor-chamber; (ii) speed of IFN-DCs in the immune-chamber (Pre-channels speed); (iii) speed of IFN-DCs in the tumor-chambers (Post-channels speed); (iv) IFN-DCs displacement, which is the covered distance from the starting point in the immune-chamber to the ending position in the tumor-chamber; and (v) DP of IFN-DC migration, which is calculated by the ratio of the Euclidean distance between the starting and the ending position of each path and the total path length. Directional persistency values close to 1 indicate a strong directional path, while values close to 0 indicate dominant Brownian motion. Speed was calculated by the ratio of total distance travelled and the duration of migration.

### Analysis of phagocytosis by Confocal Microscopy

Phagocytosis of SW620 apoptotic cells was evaluated qualitatively by confocal microscopy by placing 3D microfluidic devices on 0.17 μm coverslips and by using immunostaining procedures adapted to previously described protocols applied to 3D cultures^63^. Briefly, after a 48 h cell culture in the device, chambers and channels were washed with PBS. Then, all loading chambers were filled with 50 μl of 4% paraformaldehyde for cell fixation. After washing, specific labelling of the IFN-DCs was performed at 4 °C for 15 min, by adding 5 μl of anti-human HLA-DR-FITC Ab to the upper loading chambers. High-resolution images (800 × 800 pixel, 8 μs/pixel) were acquired with an IX83 FV1200 laser-scanning confocal microscope equipped with FluoView 4.2 software using a UPLANSAPO 20×/0.75NA objective (all from Olympus)^64^. Image stacks were processed with Imaris software for orthogonal view and 3D rendering.

### Transwell migration assay

5 × 10^5^ IFN-DCs were resuspended in RPMI-1640 containing 0.1% bovine serum albumin (BSA) and seeded in the upper chambers of 8-μm porous polycarbonate membranes of 24-well plates (Corning, NY), in the presence or absence of 10 μM AMD3100 (Sigma-Aldrich). The lower chambers were filled with supernatants (CM) from NT or RI-treated SW620 cells. After a 4 h incubation at 37 °C, IFN-DCs migrated through the filters into the lower compartments were collected and counted. The lower compartment of the control chambers contained medium alone. Each assay was performed in triplicate.

### RNA extraction, cDNA synthesis and qPCR

Total RNA was extracted from the cells using the Rneasy® Mini kit (Qiagen), according to the manufacturer’s instructions and quantified using NanoDrop ND-1000 (Thermo Scientific, USA). RNAs were reverse transcribed as previously described^8^. qPCR was performed with the DNA binding dye SYBR green (Power SYBR Green PCR master kit; Applied Biosystems) using an ABI PRISM 7900 (Applied Biosystems). qPCR were performed by using the following oligonucleotides: Homo sapiens chemokine (C-C motif) receptor 4 (CCR4), NM_005508.4, For-TGTTCACTGCTGCCTTAATCCCATC, Rev-TGGACTGCGTGTAAGATGAGCTGG; Homo sapiens chemokine (C-C motif) ligand 21 (CCL21), NM_002989.3, For-TATCCTGGTTCTGGCCTTTG, Rev- CAGCCTAAGCTTGGTTCCTG; Homo sapiens chemokine (C-C motif) receptor 2 (CCR2), transcript variant A, NM_001123041.2, For-AGAGGCATAGGGCAGTGAGA, Rev-GCAGTGAGTCATCCCAAGAG. Data were normalized to HPRT1.

### qPCR using dedicated function-tested RT-qPCR assays

Applied RT-qPCR assays for selected human gene targets were obtained as Real-Time ready Custom Panel plate for 96 reactions (Roche Diagnostics) in a dried-down format. For qPCR assays an extensive list of selected human genes is reported in Supplementary Table 3. Each well contained specific primers and a Universal ProbeLibrary Probe. The mean of expression of three different housekeeping genes (HPRT1, GAPDH andPGK1) was used to normalize the data. The qPCR assays were run in a Roche LightCycler® 480 following the manufacturer’s instructions. qPCR assays were run in at least 3 independent assays.

### Statistical analyses

Each experiment was repeated at least three times, yielding comparable results. Graphpad Prism v.5.03 (La Jolla, CA) and Microsoft Excel were used to graph the data as mean ± s.d. or s.e.m. and to calculate *P*-values using the two-tailed unpaired Student’s t-test. In all experiments, a *P*-value ≤ 0.05 was considered as statistically significant.

## Electronic supplementary material

Supplementary Info

Supplementary Movie 1

Supplementary Movie 2

Supplementary Movie 3

Supplementary Movie 4

Supplementary Movie 5
