# Supplementary material for: 3D Microfluidic model for evaluating immunotherapy efficacy by tracking dendritic cell behaviour toward tumor cells
Source: Sci Rep. 2017 Apr 24;7:1093. doi: 10.1038/s41598-017-01013-x (PMC5430848; doi:10.1038/s41598-017-01013-x)
Supplement: Supplementary file 1 — Supplementary Info [file 41598_2017_1013_MOESM1_ESM.pdf]

Supplementary Information

3D Microfluidic model for evaluating immunotherapy efficacy by tracking dendritic cell behaviour toward tumor cells

Stefania Parlato[1*], Adele De Ninno[2,4*], Rosa Molfetta[3], Elena Toschi[1], Debora Salerno[5], Arianna Mencattini[6], Giulia Romagnoli[1], Alessandra Fragale[1], Lorenzo Roccazzello[1], Maria Buoncervello[1], Irene Canini[1], Enrico Bentivegna[1], Mario Falchi[7], Francesca Romana Bertani[4], Annamaria Gerardino[4], Eugenio Martinelli[6], Corrado Di Natale[6], Rossella Paolini[3], Luca Businaro[4**] and Lucia Gabriele[1**]

[1]Department of Hematology, Oncology and Molecular Medicine, Istituto Superiore di Sanità, 00161 Rome, Italy

[2]Department of Civil Engineering and Informatic Science, University of Rome Tor Vergata, 00133 Rome, Italy

[3]Department of Molecular Medicine, Istituto Pasteur-Fondazione Cenci Bolognetti, "Sapienza" University of Rome, 00161 Rome, Italy

[4]Institute for Photonics and Nanotechnology, Italian National Research Council, 00156 Rome, Italy

[5]Center for Life Nano Science@Sapienza, Istituto Italiano di Tecnologia, 00161 Rome, Italy

[6]Department of Electronic Engineering, University of Rome Tor Vergata, 00133 Rome, Italy

[7]National AIDS Center, Istituto Superiore di Sanità, 00161 Rome, Italy

Supplementary Figures

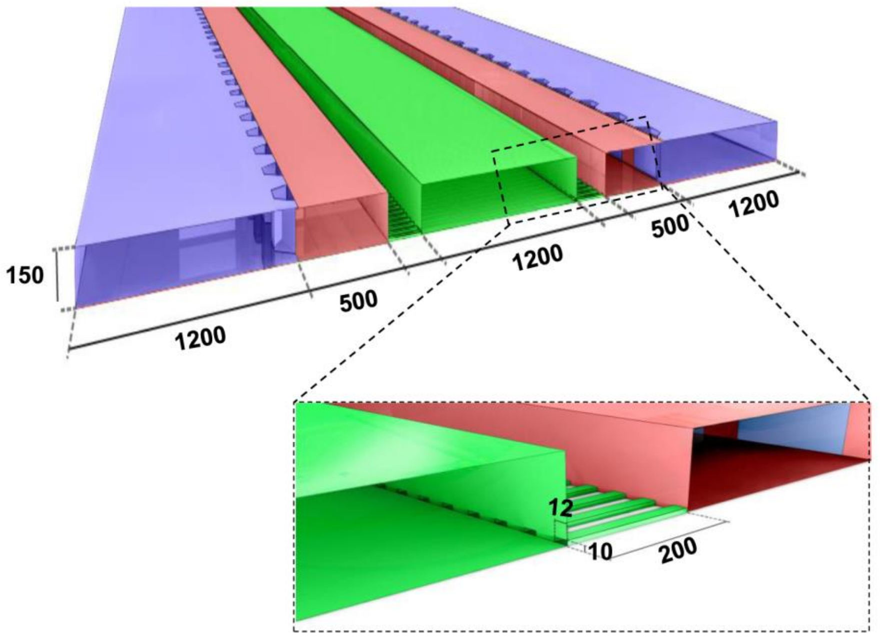

Supplementary Figure 1. Dimension of the microfluidic device. Schematic representation of a 3D section of the microfluidic platform with detailed dimensions. The boxed area represents a magnification of connecting-channels section.

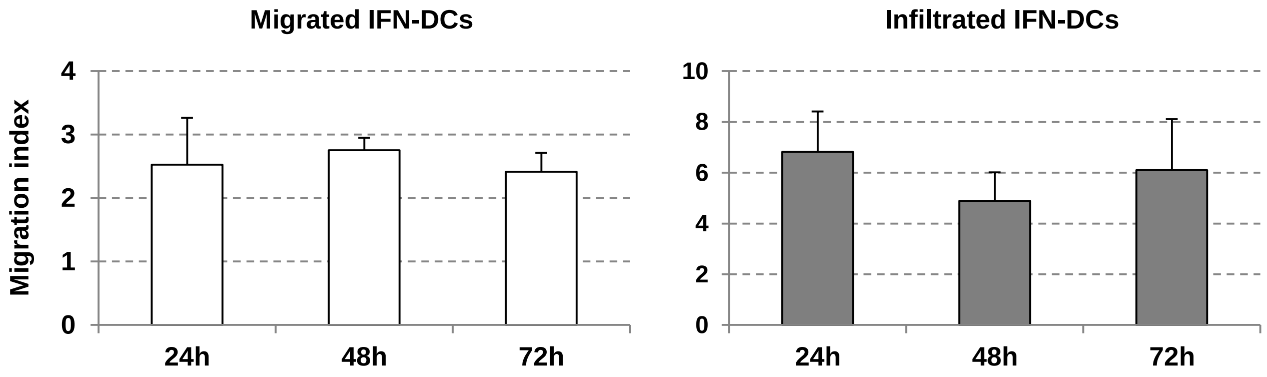

Supplementary Figure 2. Preferential migration of IFN-DCs toward RI SW620. Migration index of IFN-DCs toward RI with respect to NT SW620, at indicated time point of observation. Migration index is calculated as ratio between no. of migrating or infiltrating IFN-DCs toward RI SW620 and no. of migrating or infiltrating IFN-DCs toward NT SW620. Means ± sem are shown.

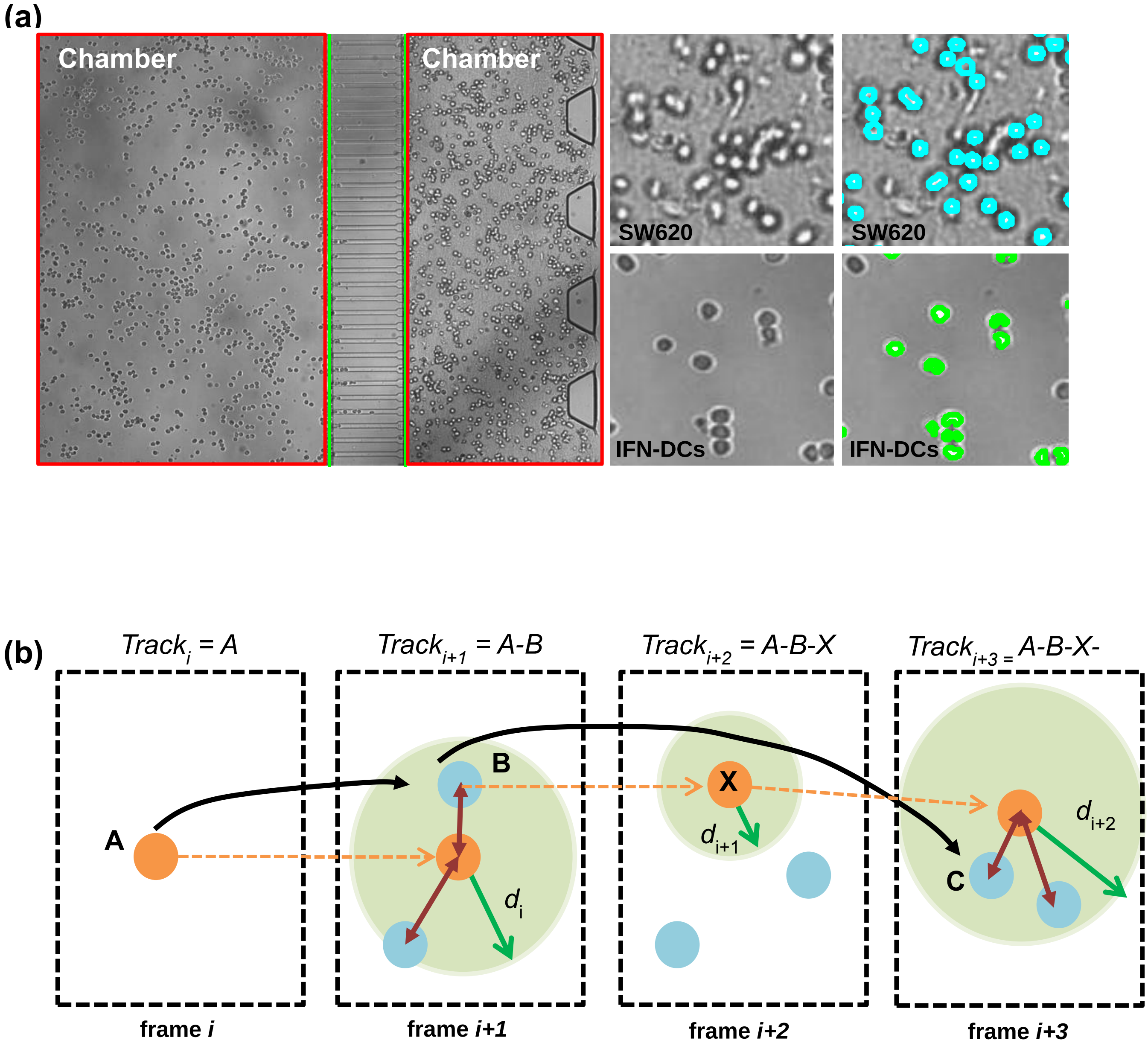

Supplementary Figure 3. Cell tracking criteria. (**a**) Identification of connecting-channels, delimited by green vertical lines, and definition of immune-chamber (chamber A) and tumor-chamber (Chamber B) by means of background elimination step (left panel) and unsupervised cell segmentation step by means of CHT algorithm (right panels). See Supplementary Methods. Right upper panels indicate SW620 cells and the corresponding segmentation results (cyan circles); the right lower panels indicate IFN-DCs and the corresponding segmentation results (green circles). (**b**) Experimental approach for the identification of moving IFN-DCs. Schematic representation of the movement detection analysis by means of consecutive frame recordings and subtraction procedure (movement map, *MP*). Boxes represent an example of four consecutive *MP* frames. Due to the

pixel-by-pixel subtraction procedure, only portion of objects moving frame-by-frame appear in a *MP* frame, except for the first *MP* frame containing the segmented cells. Object A in the *MP* frame i is linked to the nearest object falling in the region, i.e., object B in frame i+1. When no object is linked - radius d_{i+1} too small or objects too far to be linked - a not assessed position (coded by X) is assigned to the trajectory (frame i+2). The position of object B in frame i+2 is stopped and reconsidered as the starting point for the linking procedure in next frame i+3. Here, object B is linked to object C and the resulting trajectory is coded as A-B-X-C and so on (frame i+3).

(a)

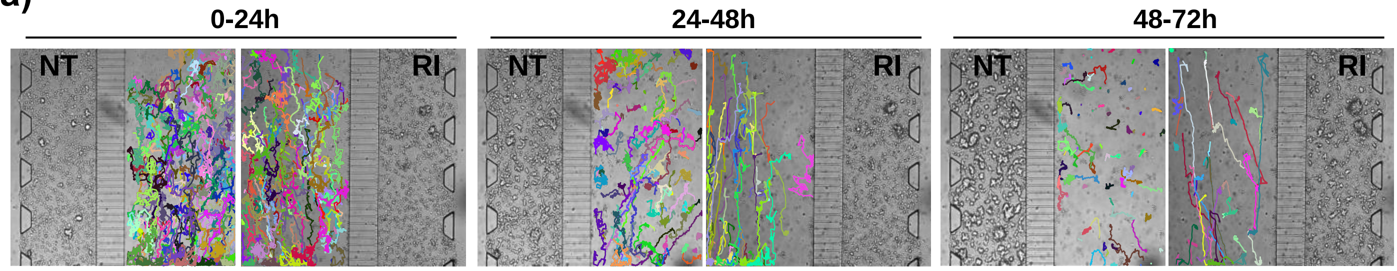

(b)

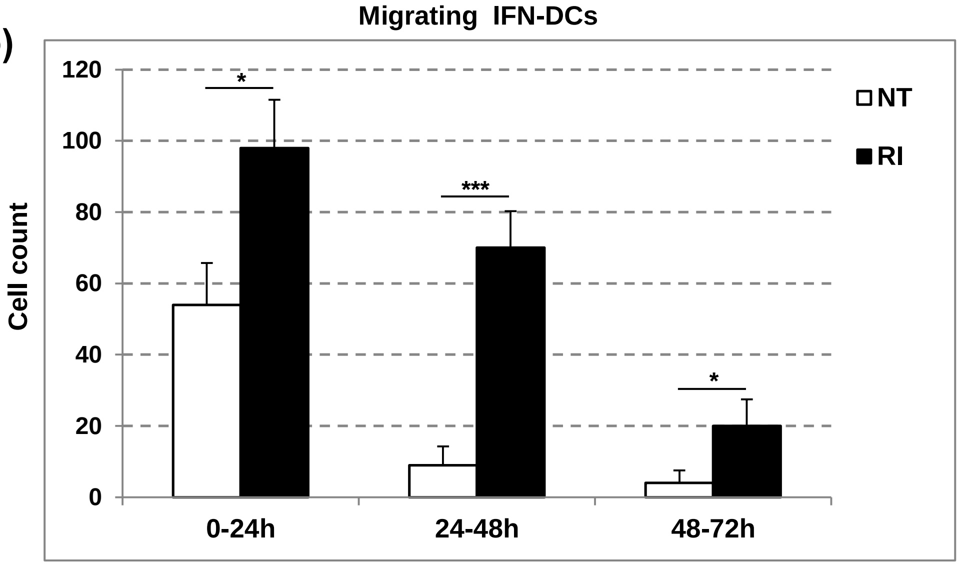

Supplementary Figure 4. (**a**) Number of IFN-DC paths identified by the automatic video processing algorithm in the immune-chamber over 0-72 h period. (**b**) Number of migrating IFN-DCs toward NT or RI SW620 manually analysed by means of ImageJ software, over 0-72 h time-lapse recording. Means ± s.d. are shown. (*P**≤0.05; ***≤0.001. Student's t-test).

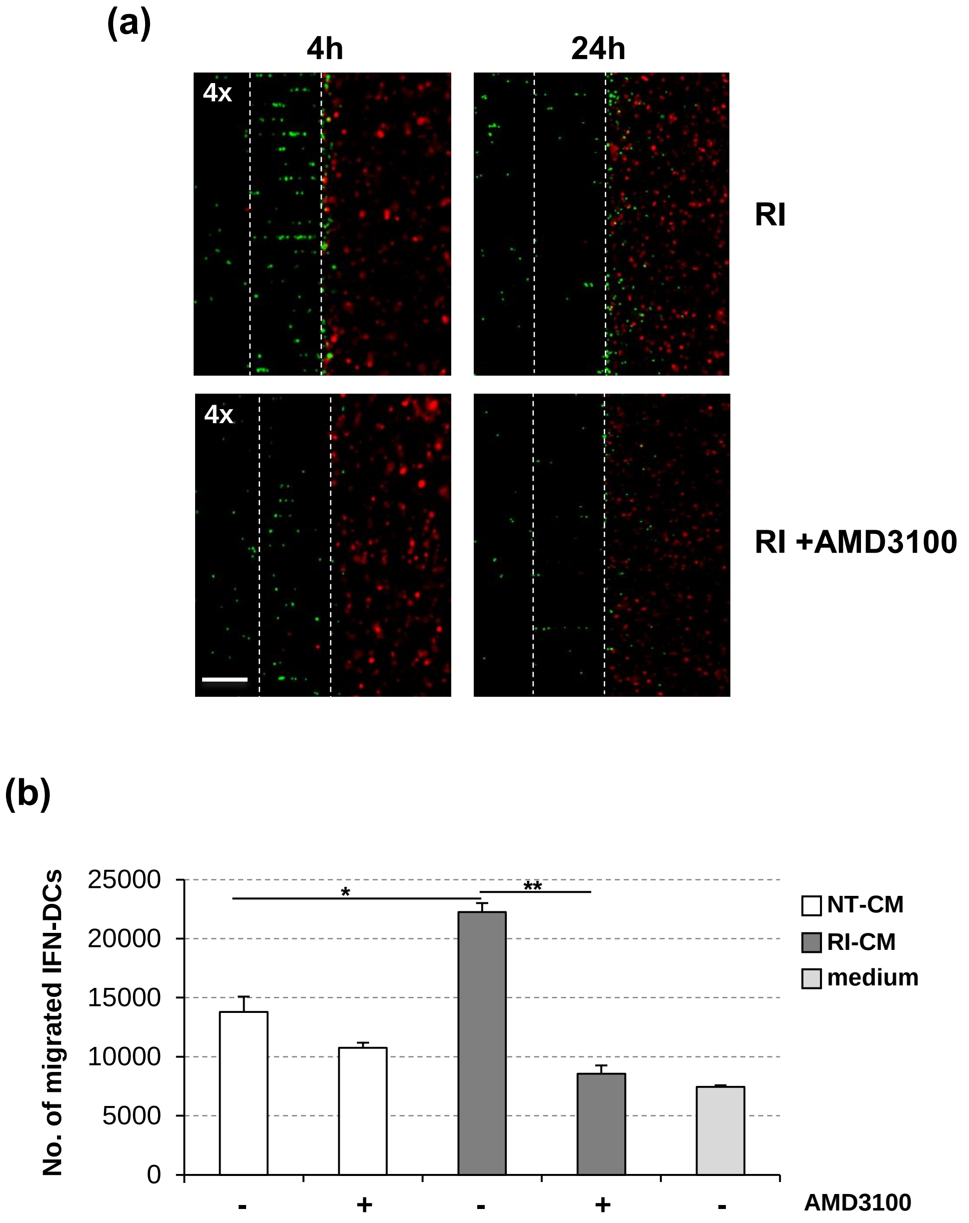

Supplementary Figure 5. Effect of CXCR4 inhibition on IFN-DC migration. (**a**) Fluorescence images, acquired using a Plan Achromat 4x/0.10NA objective, showing the migration of PKH67 green-stained IFN-DCs toward PKH26 red-stained RI SW620 (upper panels) and the effect of the CXCR4 inhibitor AMD3100 on migration (lower panels) at 4, 18 and 24 h observation time points (Scale bar, 100 μm). One representative experiment out of two is shown. (**b**) Transwell assay for the analysis of IFN-DC migration driven by conditioned medium (CM) from NT or RI-treated SW620 cells in the presence or absence of the CXCR4 inhibitor AMD3100. Cells were collected and counted after 4 h migration. Means ± s.e.m. of three independent experiments are shown. *P* *≤0.05, **≤0.01, Student's t-test.

Supplementary Table 1. Areas of immune-chamber and tumor-chamber analysed in the time-lapse experiments. Three independent experiments were performed.

	NT SW620		RI SW620	
Time (hr)	Immune-chamber (mm^2)	Tumor-chamber (mm^2)	Immune-chamber (mm^2)	Tumor-chamber (mm^2)
0-24	1.1250	0.6626	1.1341	0.6626
24-48	1.1305	0.6663	1.1159	0.6608
48-72	1.1269	0.6626	1.1105	0.6645

Supplementary Table 2. Numbers of Paths of IFN-DCs moving from immune-chamber toward tumor-chambers by crossing connecting-channels, over 0-72 h period in time-lapse experiments. Three independent experiments were performed.

	NT SW620		RI SW620	
**Time (hr)**	**Immune-chamber**	**Tumor-chamber**	**Immune-chamber**	**Tumor-chamber**
0-24	271	28	229	64
24-48	137	12	57	47
48-72	92	5	45	21

Supplementary Table 3. RealTime ready Custom Panels

Type	Assay ID	Gene Symbol H.sapiens	Alias	Description
Catalog Assay	105522	*CXCL1*	FSP, GRO1, GROa, MGSA, MGSA-a, NAP-3, SCYB1	chemokine (C-X-C motif) ligand 1 (melanoma growth stimulating activity, alpha) [Source:HGNC Symbol;Acc:4602]
Catalog Assay	111260	*CXCL14*	BMAC, bolekine, BRAK, Kec, KS1, MGC10687, MIP-2g, MIP2G, NJAC, SCYB14	chemokine (C-X-C motif) ligand 14 [Source:HGNC Symbol;Acc:10640]
Catalog Assay	103845	*CCL19*	CKb11, ELC, exodus-3, MGC34433, MIP-3b, MIP3B, SCYA19	chemokine (C-C motif) ligand 19 [Source:HGNC Symbol;Acc:10617]
Catalog Assay	110668	*CCL21*	6Ckine, CKb9, ECL, exodus-2, MGC34555, SCYA21, SLC, TCA4	chemokine (C-C motif) ligand 21 [Source:HGNC Symbol;Acc:10620]
Catalog Assay	110641	*CXCR2*	CD182, CDw128b, CMKAR2, IL8R2, IL8RA, IL8RB	chemokine (C-X-C motif) receptor 2 [Source:HGNC Symbol;Acc:6027]
Catalog Assay	104069	*CCR5*	CC-CKR-5, CCCKR5, CD195, CKR-5, CKR5, CMKBR5, FLJ78003, IDDM22	chemokine (C-C motif) receptor 5 [Source:HGNC Symbol;Acc:1606]
Catalog Assay	111317	*CCR7*	BLR2, CD197, CDw197, CMKBR7, EBI1	chemokine (C-C motif) receptor 7 [Source:HGNC Symbol;Acc:1608]
Catalog Assay	137009	*XCR1*	CCXCR1, GPR5	chemokine (C motif) receptor 1 [Source:HGNC Symbol;Acc:1625]
Catalog Assay	103807	*CXCL10*	C7, crg-2, gIP-10, IFI10, INP10, IP-10, mob-1, SCYB10	chemokine (C-X-C motif) ligand 10 [Source:HGNC Symbol;Acc:10637]
Catalog Assay	103136	*IL8*	3-10C, AMCF-I, b-ENAP, CXCL8, GCP-1, GCP1, IL-8, K60, LECT, LUCT, LYNAP, MDNCF, MONAP, NAF, NAP-1, SCYB8, TSG-1	interleukin 8 [Source:HGNC Symbol;Acc:6025]
Catalog Assay	100240	*CCL2*	GDCF-2, HC11, HSMCR30, MCAF, MCP-1, MCP1, MGC9434, SCYA2, SMC-CF	chemokine (C-C motif) ligand 2 [Source:HGNC Symbol;Acc:10618]
Catalog Assay	136209	*CCL4*	Act-2, ACT2, AT744, G-26, LAG1, MGC104418, MGC126025, MGC126026, MIP-1-beta, MIP1B, MIP1B1, SCYA2, SCYA4	chemokine (C-C motif) ligand 4 [Source:HGNC Symbol;Acc:10630]

Catalog Assay	113471	*CXCR3*	CD182, CD183, CKR-L2, CMKAR3, GPR9, IP10-R, Mig-R, MigR	chemokine (C-X-C motif) receptor 3 [Source:HGNC Symbol;Acc:4540]
Catalog Assay	143194	*CCR6*	BN-1, C-C CKR-6, CC-CKR-6, CCR-6, CD196, CKR-L3, CKRL3, CMKBR6, DCR2, DRY-6, DRY6, GPR-CY4, GPR29, GPRCY4, STRL22	chemokine (C-C motif) receptor 6 [Source:HGNC Symbol;Acc:1607]
Catalog Assay	143341	*CCR9*	CDw199, GPR-9-6, GPR28	chemokine (C-C motif) receptor 9 [Source:HGNC Symbol;Acc:1610]
Catalog Assay	141070	*CXCR4*	CD184, D2S201E, FB22, fusin, HM89, HSY3RR, LAP3, LCR1, LESTR, NPY3R, NPYR, NPYRL, NPYY3R	chemokine (C-X-C motif) receptor 4 [Source:HGNC Symbol;Acc:2561]
Catalog Assay	144645	*CXCL12*	IRH, PBSF, SCYB12, SDF-1a, SDF-1b, SDF1, SDF1A, SDF1B, TLSF, TLSF-a, TLSF-b, TPAR1	chemokine (C-X-C motif) ligand 12 [Source:HGNC Symbol;Acc:10672]
Catalog Assay	104231	*CXCL9*	CMK, crg-10, Humig, MIG, SCYB9	chemokine (C-X-C motif) ligand 9 [Source:HGNC Symbol;Acc:7098]
Catalog Assay	110753	*CCL20*	CKb4, exodus-1, LARC, MIP-3a, MIP3A, SCYA20, ST38	chemokine (C-C motif) ligand 20 [Source:HGNC Symbol;Acc:10619]
Catalog Assay	113395	*CCL5*	D17S136E, MGC17164, RANTES, SCYA5, SISd, TCP228	chemokine (C-C motif) ligand 5 [Source:HGNC Symbol;Acc:10632]
Catalog Assay	144064	*CCR1*	CD191, CKR-1, CKR1, CMKBR1, HM145, MIP1aR, SCYAR1	chemokine (C-C motif) receptor 1 [Source:HGNC Symbol;Acc:1602]
ReferenceGene	102079	*HPRT1*	HGPRT, HPRT	hypoxanthine phosphoribosyltransferase 1 [Source:HGNC Symbol;Acc:5157]
ReferenceGene	141139	*GAPDH*	G3PD, GAPD, MGC88685	glyceraldehyde-3-phosphate dehydrogenase [Source:HGNC Symbol;Acc:4141]
ReferenceGene	102083	*PGK1*	MGC117307, MGC142128, MGC8947, MIG10, PGKA	phosphoglycerate kinase 1 [Source:HGNCSymbol;Acc:8896]

Supplementary Methods

Cell tracking analysis. The identification of cells to be tracked relied on image analysis performed by a novel unsupervised image processing algorithm described in Section Methods. *Background elimination step*. This step is required to identify and isolate barriers, channels and chambers that appear in each frame. We assume that these parameters are fixed thus this step is performed only in the first frame in order to speed the process. A line detector based on the Hough transform is applied to the entire image in order to extract the connecting-channels and define the immune-chamber loaded with IFN-DCs and the tumor-chambers filled with SW620 cells. *Segmentation step.* This step allowed to localize IFN-DCs in the immune-chamber and SW620 cells in the tumor-chambers. Cells were visualized at 1.33 μm/px spatial resolution. SW620 cells were modelled as circles and defined by an estimated centre and a radius (mean radius 4.87 μm ± s.d.1.18 μm). Segmentation of SW620 cells was performed by applying the Circular Hough Transform (CHT) algorithm, a modified version of the standard Hough transform for detecting linear barrier, able to detect the centre of circular objects and the corresponding radius using the concept of accumulation matrix. Image up-sampling with a scale factor of 4 through bilinear interpolation and parallel processing are implemented to improve performance of object detection and simultaneously keep an acceptable computational cost. Assuming that SW620 cells into type I collagen matrix perform limited movements, the segmentation step applied on the first image frame was used for the entire video sequence. Since IFN-DCs and SW620 cells showed different features (i.e. dark nucleus, light borders and the ability to assume elongated shape for moving IFN-DCs; light nucleus, dark border and circular shape for SW620 cells), we selected a different segmentation algorithm for the two cell population. In particular, we used standard blob-detector based on multiscale Laplacian of Gaussian (LoG) filtering and adaptive image thresholding techniques to segment IFN-DCs. The

latter performs binarization of the processed images by using a threshold automatically selected in order to obtain minimum area dispersion over the segmented objects. Due to the very high number of cells in each frame of the video, manual delineation for segmentation assessment could not be performed. For this reason, segmentation accuracy was assessed through a phantom sequence of images generated to simulate the real scenario. In particular, cell average intensity, size, and density were estimated from the actual video sequence and used in the phantom video setting. A sequence of 100 frames was generated in order to simulate a left chamber with immune cells and a right chamber with cancer cells. Immune cells (4 pixels radius, dark intensity) occupied 1% of the left area (178 cells) whereas cancer cells (4 pixels radius, bright intensity) occupied the 12% of the right area (1373 cells). Cells were randomly located in their chamber. Gaussian noise was also added to each frame in order to simulate image degradation due to gel and background effects. Segmentation accuracy was evaluated in terms of the standard accuracy metrics, True Positive Volume Fraction (TPVF) and False Positive Volume Fraction (FPVF) (Udupa J.K. et al. Comput. Med. Imaging Graph., 2006). Mean and s.e.m. values of TPVF (FPVF) for 100 random phantom videos resulted in 0.998 ± 0.002 (<1e-8 ± < 1e-8) for the immune cells and 0.815 ± 0.008 (0.008 ± < 1e-4) for the cancer cells. *Linking step*. First, a preliminary detection of movements by standard consecutive frame recording and subtraction procedure, so-called *movement map* (*MP*), was performed to avoid the linking of IFN-DCs with totally stationary objects (i.e., objects that disappear after image subtraction). In the *MP*, each frame, coded by centroid (x,y) coordinates, is linked to the next frame by the nearest object leading to the construction of xy domain trajectory, denoted by the position of vector (x(t),y(t)). The pipeline of the algorithm is in Supplementary Figure 3b.

RT-qPCR assays. The Universal ProbeLibrary Probe (UPL) contained in the Applied RT-qPCR assays (Roche Diagnostics) is a short FAM-labelled hydrolysis probe containing locked nucleic acid (LNA). The UPL is based on only 165 short hydrolysis probes, labelled at the 5′ end with

fluorescein and at the 3′ end with a dark quencher dye. Each probe can cover up to several thousand binding sites and the whole set of 165 probes thereby covers ~99.9% of the human transcriptome. Incorporation of locked nucleic acids into the probe sequence increased the thermal stability and discriminative power.

Supplementary Movie Legends

Supplementary Movie 1. Time-lapse recording of IFN-DC migration toward NT SW620. IFN-DCs were loaded into the immune-chamber and allowed to migrate towards tumor-chamber filled with NT or RI-treated SW620 cells through connecting-channels. The movie shows the migratory behaviour of IFN-DCs toward NT SW620 over 72 h period.

Supplementary Movie 2. Time-lapse recording of IFN-DC migration toward RI SW620. IFN-DCs were loaded into the immune-chamber and allowed to migrate toward tumor-chamber filled with NT or RI-treated SW620 cells through connecting-channels. The movie shows the migratory behaviour of IFN-DCs toward RI SW620 over 72 h period.

Supplementary Movie 3. Time-lapse recording of IFN-DC migration toward NT SW620 or RI SW620. Supplementary Movies 1 and 2 were edited using the Sony Vegas 8.0 software (Sony Creative Software, Madison, WI, USA).

Supplementary Movie 4. 3D movie recording PKH-67-labelled IFN-DCs (green) moving in 3D tumor space and interacting with RI-treated-PKH-26-labelled SW620 cells (red). Wide field image stacks (100 images of 1μm Z-step size) were acquired every 15 minutes and movie was realized with Imaris software. 3D reconstruction is shown.

Supplementary Movie 5. 3D movie recording the interaction of PKH-67-labelled IFN-DCs (green) with RI-treated-PKH-26-labelled SW620 cells (red). In the central area of movie, as indicated by the arrow, is depicted one IFN-DC performing phagocytosis of a red apoptotic body derived from RI-treated SW620 cell. Wide field image stacks (100 images of 1μm Z-step size) were acquired every 15 minutes and movie was realized with Imaris software. Orthogonal xy and yz views of the phagocytic event are shown.
